# An annotated catalogue of the types of Chrysididae (Hymenoptera) at the Swedish Museum of Natural History, Stockholm, with brief historical notes

**DOI:** 10.3897/zookeys.495.9356

**Published:** 2015-04-08

**Authors:** Paolo Rosa, Hege Vårdal

**Affiliations:** 1 Via Belvedere 8/d I-20881 Bernareggio (MB), Italy; 2 Swedish Museum of Natural History, Department of Entomology, Box 50007, SE-104 05 Stockholm, Sweden

**Keywords:** Chrysididae, catalogue, neotype designation, lectotype designation

## Abstract

A critical and annotated catalogue of 72 types of Chrysididae (Hymenoptera) belonging to 53 species and subspecies housed in the Swedish Museum of Natural History is given. The lectotypes of *Chrysis diversa* Dahlbom, 1845, *Chrysis soror* Dahlbom, 1854, *Chrysura sulcata* Dahlbom, 1845 and *Holopyga amoenula* Dahlbom, 1845 are designated. The previous lectotype of *Chrysis diversa* Dahlbom, 1845 is set aside. Five new synonymies are proposed: Chrysis elegans var. smaragdula Trautmann, 1926 (currently Chrysis elegans ssp. interrogata Linsenmaier, 1959 repl. name for *smaragdula* Trautmann, *nec* Fabricius, 1775), **syn. n.** of *Chrysis confluens* (Dahlbom, 1845); *Chrysis eximia* Mocsáry, 1889, **syn. n.** of *Chrysis poecila* Mocsáry, 1889; *Chrysis pyrrhina* Dahlbom, 1845, **syn. n.** of *Chrysis erythromelas* Dahlbom, 1845; *Chrysis separata* Trautmann, 1926, **syn. n.** of *Chrysis lateralis* Dahlbom, 1845; *Chrysis sicula* Abeille de Perrin, 1877, **syn. n.** of *Chrysis erythromelas* Dahlbom, 1845. *Chrysis serena* Radoszkowski, 1891 is the first available name for *Chrysis pyrrhina sensu auctorum*. *Chrysis erythromelas* Dahlbom, 1845 is revaluated as valid species. The neotype of *Chrysis inaequalis* Dahlbom, 1845 is designated in the Linsenmaier collection (NMLS). Illustrations of 34 types are given.

## Introduction

The Chrysididae collection in the Swedish Museum of Natural History (NHRS) is an important historical collection in Europe that includes several types described by Dahlbom and other authors. It is divided in three parts: the general collection, the Swedish collection and the type collection. A few specimens (294 specimens) of Chrysididae can be found in separate historical collections (Boheman’s collection).

The general collection consists of 15 drawers that were reorganized by the first author in 2012 in taxonomical and alphabetical order *sensu* Kimsey and Bohart (1991) and it includes about 1700 specimens. The Swedish collection consists of 19 drawers and 1762 specimens belonging to about 50 taxa. All the type specimens were labelled with red type labels and transferred to the type collection, which currently includes 72 types belonging to 53 species and subspecies: 30 holotypes, 20 paratypes, 7 syntypes, 6 lectotypes and 9 paralectotypes. Unfortunately, the original identification labels by Dahlbom are lost, probably removed after a subsequent reorganization of the collection in the nineteenth century. For this reason we encountered some difficulties in identifying some original types (e.g. *Platycelia ehrenbergi* and *Stilbum wesmaeli*).

Dahlbom (1845) did not list all the examined specimens, but he used different Latin words related to the frequency at which he encountered the examined species: *vulgatissima* (very common), *vulgar* (common), *freq.* (= *frequentes*, frequent), *pl. min. freq.* (*plus or minus frequentes*, more or less frequent), *pass*. (= *passim*, literally ‘here and there’), *rar.* (= *rarus*, used when he examined few specimens) and *rariss*. (= *rarissima*, when he examined only one specimen). These characterisations were taken into consideration when studying the type material. At that time, the Code of Zoological Nomenclature was not yet published, and Dahlbom (1845, 1854) did not follow the “Principle of Priority”. In some cases, he changed the priority of species previously described. These changes led to confusion among the following authors, as shown in the remarks (e.g. *Chrysis mediocris*, *Hedychridium cupreum*, *Holopyga amoenula*). In other cases he changed the original description, after the examination of further material (e.g. *Chrysis sulcata*).

The present paper is mainly focused on the type material described by Dahlbom, but also includes other Chrysidid types described by authors after Dahlbom and housed at the Swedish Museum of Natural History (NHRS). Cameron (1910) and Hammer (1950) described some species and dedicated two new species to Yngve Sjöstedt, the professor and curator of the entomology department of the NHRS: *Chrysis sjostedti* Cameron and *Cleptes sjostedti* Hammer. Some paratypes were donated by Linsenmaier (1959a), who was in contact and exchanged several specimens with Stellan Erlandsson and the Gaunitz family. In the 1960s the museum loaned some exotic specimens to the Swiss entomologist Walter Linsenmaier, who described a new species (*Chrysis tenuimediata* Linsenmaier, 1968). A great part of that loan remained unidentified and was sent back to the Museum after Linsenmaier’s death. The Finnish entomologist Erikki Valkeila (1971) deposited the holotype of *Chrysis corusca* and the paratype of *Chrysis scintillans* here. Valkeila was very active and identified many specimens in the NHRS Chrysididae collection. In the 1980s Bohart borrowed some African types, and kindly deposited some paratypes of Nearctic species. It is unclear how two types by Balthasar (1957) arrived in the collection.

Anders Gustaf Dahlbom was born in Herrberga parish in Östergötland County on March 3, 1806. From his father, the surgeon Anders Dahlbom, he inherited a strong interest in insects (Svenskt Biografiskt Lexikon 2013). He matriculated at Lund University in 1825, studied natural history, medicine and pharmacology and completed his master’s degree (Dahlbom 1829), with a thesis on Chrysididae (*Monographia Chrysidum Sveciæ*). He became a docent of natural history in 1830 in Lund and from 1843 lecturer in entomology as well as curator of the entomological collections at the Museum of Zoology at Lund University. In 1857, two years before he died, Dahlbom was appointed professor (Dal 1996). Dahlbom was a pioneer in applied entomology and wrote a handbook for farmers and naturalists about common benefits and potential problems with the Scandinavian insects that can be found in and around a house or farm (Dahlbom 1838). However, most of his works are on systematic entomology and are characterized by careful descriptions and sharp-eyed observations (Svenskt Biografiskt Lexikon 2013). He took part in several entomological research journeys with his teacher Johan Wilhelm Zetterstedt in northern Sweden and abroad.

Dahlbom had the opportunity to visit some of the museums that were the most important in Europe at that time: Berlin (MNHU), Copenhagen (ZMUC), London (BMNH), Paris (MNHN), and his types are currently found in Berlin (MNHU), Copenhagen (ZMUC), Lund (MZLU), Stockholm (NHRS), Turin (MRSN) and Vienna (MHNW). He published his observations and studies on Chrysididae in four publications: *Exercitationes Hymenopterologicae, Monographia Chrysididum Sveciae* (Dahlbom 1831), *Dispositio Methodica Specierum Hymenopterorum. Particula II – Chrysis in sensu Linnæano* (Dahlbom 1845), *Syd-Africanska Chrysides* (Dahlbom 1850), *Hymenoptera europaea praecipue borealia* (Dahlbom 1854). The latter is considered a landmark in the study of Chrysididae. For the first time he provided keys to genera and species and an attempt to organize all the known information on Chrysidids at that time. In total he described 213 new species (Dahlbom 1854) of which more than 150 are still valid (Kimsey and Bohart 1991), and his descriptions were used as models for that time. Dahlbom examined Fabricius’ types deposited at Kiel (ZMUC) and in Vienna (MHNW), Klug’s types in Berlin (MNHU) and Spinola’s types from his private collection (MRSN, Rosa and Xu 2015). Dahlbom passed away on May 3, 1859, in Lund. Most of his large collection, his library, a rich archive of correspondence with international and national researchers, and a catalogue of the collections and their history were donated to the entomological collections in Lund (MZLU) (Svenskt Biografiskt Lexikon 2013).

## Material and methods

Terminology and classification of the genera follows Kimsey and Bohart (1991). Classification of species follows Fauna Europaea (Rosa and Soon 2012), Linsenmaier (1959, 1968, 1987, 1997a, 1997b, 1999), Rosa (2006), Van der Smissen (2010) and Móczár (1998a, b), for the genus *Cleptes*. These works have been taken in consideration also for the reorganization of the general collection. The 4^th^ edition of the International Code of Zoological Nomenclature (ICZN), in effect since 1^st^ January 2000, has been applied to the present work.

The type list is arranged alphabetically and the following data are given: name of the species and of the author, the complete reference of the description, type locality, current systematic placement, category of the type, number and sex of specimens, complete label, in which handwritten text is given in italics; labels are separated from each other by square brackets; a stroke marks the end of a line. The state of preservation is given only in case of damaged types.

Only selected types were illustrated, such as the newly designated neotype and lectotypes. Pictures of the types were taken with Nikon D-80 connected to the stereomicroscope Togal SCZ and stacked with the software Combine ZP (by Paolo Rosa); the white calibration of the photocamera was applied to reduce the blue effect of the neon light of the Togal microscope. Two pictures were taken with Canon EOS 7D combined with the software Zerene Stacker (“HV” photos = by Hege Vårdal).

All the chrysidid types housed at the NHRS were labelled with NHRS-HEVA catalogue numbers and databased in the DINA-system used by several Swedish natural history collections. This data is presented on Naturarv which is the Search Portal for Natural History Collections in Sweden (www.naturarv.se). GBIF harvest data from this system on a regular basis. High resolution photographs of the types presented in this paper will be uploaded on the database of biological images Morphbank (www.morphbank.net).

Other specimens examined or discussed are deposited in the following institutions:

BME Bohart Museum of Entomology, University of California, Davis, USA.

BMNH The Natural History Museum, London, United Kingdom.

HNHM Hungarian Natural History Museum, Budapest, Hungary.

ISEA–PAS Invertebrate collections of the Institute of Systematics and Evolution of Animals, Polish Academy of Sciences in Krakow, Poland.

MNHN National Museum of Natural History, Paris, France.

MNHU Museum of Natural History of the Humboldt-Universität, Berlin, Germany.

MRSN Regional Museum of Natural Science, Turin, Italy.

MZH Finnish Museum of Natural History, University of Helsinki; Helsinki, Finland.

MZLU Lund Zoological Museum, University of Lund, Sweden.

NHMW Natural History Museum, Vienna, Austria.

NHRS Swedish Museum of Natural History, Stockholm, Sweden.

NMLS Natur-Museum, Luzern, Switzerland.

NMPC National Museum of Natural History, Prague, Czech Republic.

ZMUC Zoological Museum, University of Copenhagen, Denmark.

ZMUK Zoological Museum, University of Kiel, Germany.

## Catalogue of the types in NHRS

### Argochrysis albicornis

Taxon classificationAnimaliaHymenopteraChrysididae

Bohart, 1982

Argochrysis albicornis : Bohart (in Bohart & Kimsey) 1982: 189.

#### Type locality.

U.S.A. (holotype from Borrego Valley, San Diego Co., California; paratypes: 44 ♂♂ and 58 ♀♀ form California and Nevada).

#### Paratype 1 ♂.

[1,000 Palms Cyn., Cal. Riverside Co. IV-9-1964] [R.M. Bohart collector] [Paratype Argochrysis albicornis ♂ *R.* M. Bohart] <red label> [NHRS-HEVA000001057].

#### Paratype 1 ♀.

[Calif *2* mi *E* Lone Pine Inyo Co. V-19-1970] [E.E. Grissell Colr] [Paratype Argochrysis albicornis ♀ R.M. Bohart] <red label> [NHRS-HEVA000001058].

#### Remarks.

The holotype is deposited at the BME.

#### Current status.

*Argochrysis albicornis* Bohart, 1982.

### Argochrysis armilla

Taxon classificationAnimaliaHymenopteraChrysididae

Bohart, 1982

Argochrysis armilla : Bohart (in Bohart & Kimsey) 1982: 189.

#### Type locality.

U.S.A. (holotype from Sagehen Creek, Nevada Co., California; paratypes 42 ♂♂ and 41 ♀♀ from the same locality).

#### Paratype 1 ♂.

[Sahegen Crk Cal. Nevada Co. VI 25 1966] [ R.L. Brumley Coll.] [Paratype Argochrysis armilla ♂ R.M. Bohart] <red label> [NHRS-HEVA000001063].

#### Paratype 1 ♀.

[Sahegen Crk Nevada Co. Cal. VII 13 68] [ RM Bohart Colr.] [Paratype Argochrysis armilla ♀ R.M. Bohart] <red label> [NHRS-HEVA000001064].

#### Remarks.

The holotype is deposited at the BME.

#### Current status.

*Argochrysis armilla* Bohart, 1982.

### Argochrysis litura

Taxon classificationAnimaliaHymenopteraChrysididae

Bohart, 1982

Argochrysis litura : Bohart (in Bohart & Kimsey) 1982: 193.

#### Type locality.

U.S.A. (holotype from Tanbark Flat, Los Angeles Co., California; paratypes 34 ♂♂ and 99 ♀♀ from Arizona, California and Idaho).

#### Paratype 1 ♀.

[Arroyo Seco Camp Calif. Monterey Co. V-15-1973] [C. Goodpasture Colr] [Paratype Argochrysis ♀ litura R.M. Bohart] <red label> [NHRS-HEVA000001096].

#### Remarks.

The holotype is deposited at the BME.

#### Current status.

*Argochrysis litura* Bohart, 1982.

### Ceratochrysis concava

Taxon classificationAnimaliaHymenopteraChrysididae

Bohart, 1982

Ceratochrysis concava : Bohart (in Bohart & Kimsey) 1982: 172.

#### Type locality.

U.S.A. (holotype from Whitewater, Riverside Co., California; paratypes 20 ♂♂ and 32 ♀♀ from Arizona, California, Nevada).

#### Paratype 1 ♂.

[Mt. Diablo Cal. V-12-39] [G.E. Bohart Collector] [Paratype Ceratochrysis concava ♂ R. Bohart] <red label> [NHRS-HEVA000001068].

#### Paratype 1 ♀.

[Mt. Diablo, Cal. V-16-40] [J.W. MacSwain Collector] [Paratype Ceratochrysis concava ♀ R. Bohart] <red label> [NHRS-HEVA000001069].

#### Remarks.

The holotype is deposited at the BME.

#### Current status.

*Ceratochrysis concava* Bohart, 1982.

### Ceratochrysis minata

Taxon classificationAnimaliaHymenopteraChrysididae

Bohart, 1982

Ceratochrysis minata : Bohart (in Bohart & Kimsey) 1982: 177.

#### Type locality.

U.S.A. (holotype from Davis, California; paratypes 34 ♂♂ and 30 ♀♀ from Alberta, California, Colorado, Idaho, Nevada, Nebraska, New Mexico, Oregon, Texas and Wyoming).

#### Paratype ♂.

[Tracy, Calif. San Joaquin Co. *V-26* 19*49*] [J.W. MacSwain Collector] [Paratype Ceratochrysis minata ♂ R. Bohart] <red label> [NHRS-HEVA 000001109].

#### Paratype ♀.

[Tracy, Calif. San Joaquin Co. *VI-3* 19*49*] [J.W. MacSwain Collector] [Paratype Ceratochrysis minata ♀ R. Bohart] <red label> [NHRS-HEVA000001110].

#### Remarks.

The holotype is deposited at the BME.

#### Current status.

*Ceratochrysis minata* Bohart, 1982.

### Chrysis bohemanni

Taxon classificationAnimaliaHymenopteraChrysididae

Dahlbom, 1845

Plate 1

Chrysis Bohemanni : Dahlbom 1845: 12.

#### Type locality.

South Africa: “*Port Natal*”.

#### Holotype ♀

(not ♂): [Caffraria] [J. Wahlb.] [Type] [*Bohemani* (sic) *Dahlb.*] [275 *82*] <red label> [NHRS-HEVA000001065].

**Plate 1.**
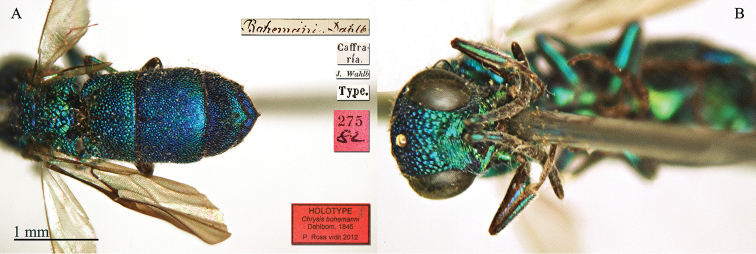
*Chrysis bohemanni* Dahlbom, 1845, holotype. **A** Metasoma, dorsal view **B** head, frontal view.

#### Remarks.

The type is a female, with the tip of the ovipositor visible. The species is dedicated to Carl Henrik Boheman (1796–1868) a Swedish entomologist. Therefore the correct name should be *bohemani* and not *bohemanni*. However, according to the Code (ICZN 1999: Article 32.5.1) in the original publication there is no clear evidence of an inadvertent error; moreover (ICZN 1999: Article 32.5.1.1), at the end of the same publication (Dahlbom 1845), a *corrigendum* is given including the correction of the name *Scönherri* in *Schönherri*, but not the correction of the name *bohemanni*. Furthermore, in the following volume (Dahlbom 1854), Carl Henrik Boheman is cited in the introduction and in the text, but Dahlbom went on using the name *Chrysis bohemanni*, fixing the wrong spelling, which is in current use (Bohart 1988; Madl and Rosa 2012; Strumia 2009).

#### Current status.

*Trichrysis bohemanni* (Dahlbom, 1845) (transferred by Bohart 1988: 349).

### Chrysis ciscirtana

Taxon classificationAnimaliaHymenopteraChrysididae

Linsenmaier, 1959

Chrysis ciscirtana : Linsenmaier 1959: 97.

#### Type locality.

Palestine.

#### Paratype 1 ♂.

[Jerusalem *5.V.43* Palestina Houska lgt.] [*Paratype* Chrysis L. ciscirtana *Lins.*♂ Linsenmaier det. 59] <handwritten in red> [NHRS-HEVA000001067].

#### Remarks.

The holotype is deposited in the Linsenmaier collection at the NMLS.

#### Current status.

*Chrysura ciscirtana* (Linsenmaier, 1959) (transferred by Kimsey and Bohart 1991: 487).

### Chrysis corusca

Taxon classificationAnimaliaHymenopteraChrysididae

Valkeila, 1971

Plate 2

Chrysis corusca : Valkeila 1971: 84.

#### Type locality.

Sweden: “Nrk. Åsbro Lerbäck”.

#### Holotype ♀.

[Sweden Närke Lerbäck, Åsbro 19*68* G. Hallin] [390 *81*] <red label> [Chrysis ♀ corusca *n.sp.* det. E. Valkeila – *69 Holotypus*] [NRM Sthlm Loan 2571/08] [Naturhistoriska Riksmuseet Stockholm Loan no 1483/96] [Chrysis ♀ schencki *Lins.* det. O. Niehuis 1997] [NHRS-HEVA000001070].

**Plate 2.**
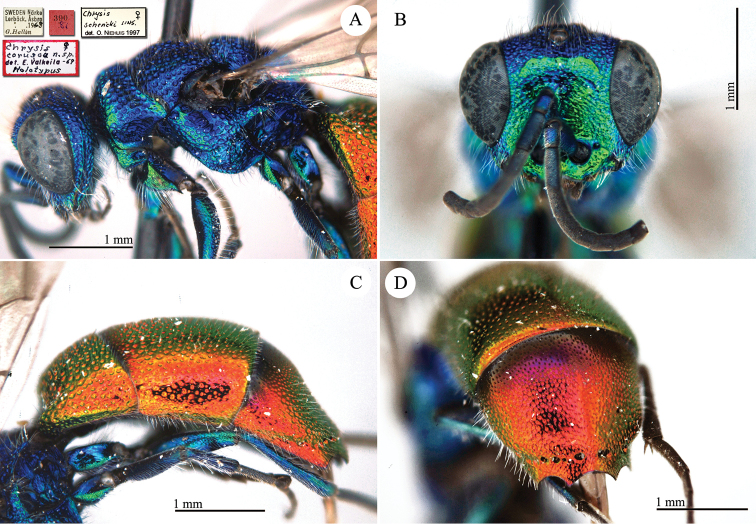
*Chrysis corusca* Valkeila, 1971, holotype. **A** Head and mesosoma, lateral view **B** head, frontal view **C** metasoma, lateral view **D** third metasomal tergite, dorso-lateral view.

#### Remarks.

For a very long time *Chrysis corusca* remained an enigmatic species. Linsenmaier (1987, 1997a) did not even cite it in his revisional works on the European species. Also the most important European revisions or checklists published in the 1990s (Kunz 1994; Mingo 1994; Strumia 1995) did not include *Chrysis corusca*. Kimsey and Bohart (1991: 400) were the first authors to include *Chrysis corusca* in a catalogue with the status of valid species. Diagnostic characteristics were cited in the original description, Niehuis (2000: 184) found other better and usable characteristics, and later listed *Chrysis corusca* as a valid species widely distributed in Germany (Niehuis 2001: 120). A detailed morphological analysis of this species was finally provided by van der Smissen (2010: 69) in her monographical work on the *Chrysis ignita* group. Soon and Saarma (2011) included *Chrysis corusca* in their molecular analysis. The distribution of this species is still poorly known and related to central and north European countries (Paukkunen et al. 2014). However we do believe that *Chrysis corusca* could have a wide distributional range and that data are missing because of misidentifications with other species within the *Chrysis ignita* species group (Rosa et al. 2013).

In the original description Valkeila listed 3 females (holotype and 2 paratypes) from Närke Lerbäck, Åsbro (leg. G. Hallin). At the moment only the holotype is present in the general collection. The two paratypes are in Gunnar Hallin’s private collection, which is scheduled for donation to the NHRS (H. Vårdal, pers. comm.).

#### Current status.

*Chrysis corusca* Valkeila, 1971.

### Chrysis dalmanni

Taxon classificationAnimaliaHymenopteraChrysididae

Dahlbom, 1845

Plate 3

Chrysis Dalmanni : Dahlbom 1845: 12.

#### Type locality.

unknown.

#### Holotype ♀.

[Mus. Payk.] [Type] [NHRS-HEVA000001071].

**Plate 3.**
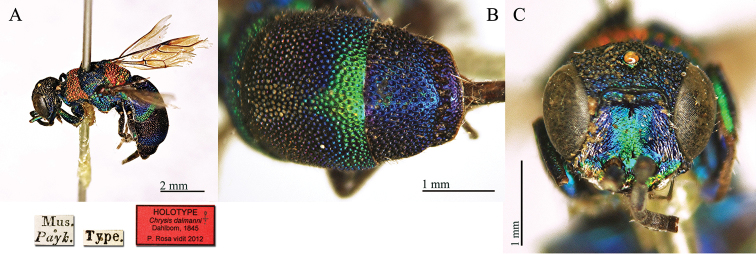
*Chrysis dalmanni* Dahlbom, 1845, holotype. **A** Habitus, dorso-lateral view **B** second and third metasomal tergites, dorsal view **C** head, frontal view.

#### Remarks.

*Chrysis dalmanni* is an Afrotropical species, known from South Africa (Mocsáry 1902b: 543; Edney 1952: 432; Kimsey and Bohart 1991: 402); Lesotho is also mentioned, but without precise locality (Madl and Rosa 2012: 29). The species is dedicated to Johan Wilhelm Dalman (1787–1828), a Swedish physician and a naturalist interested in entomology and botany. Similarly to the case of *Chrysis bohemanni*, the correct spelling should be *dalmani* and not *dalmanni*. However, also in this case (Dahlbom 1845, 1854) it is clear Dahlbom’s intention to double the final “n”, making the original surname with a German appearance.

#### Current status.

*Chrysis dalmanni* Dahlbom, 1845.

### Chrysis delicatula

Taxon classificationAnimaliaHymenopteraChrysididae

Dahlbom, 1850

Plate 4

Chrysis delicatula : Dahlbom 1850: 138.

#### Type locality.

South Africa, Natal province.

#### Holotype ♀.

[Caffraria] [J. Wahlb] [Type] [*Chrysis delicatula Dahlb.*] [Typus] <red label> [268 *82*] <red label> [NHRS-HEVA000001072].

**Plate 4.**
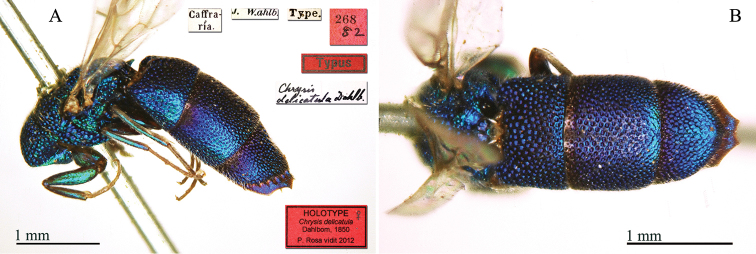
*Chrysis delicatula* Dahlbom, 1850, holotype. **A** Habitus, lateral view **B** metasoma, dorsal view.

#### Remarks.

The type is damaged, the head is missing.

#### Current status.

*Chrysis delicatula* Dahlbom, 1850.

### Chrysis diversa

Taxon classificationAnimaliaHymenopteraChrysididae

Dahlbom, 1845

Plate 5

Chrysis diversa : Dahlbom 1845: 13.

#### Type locality.

Egypt.

#### Lectotype

**Plate 5.**
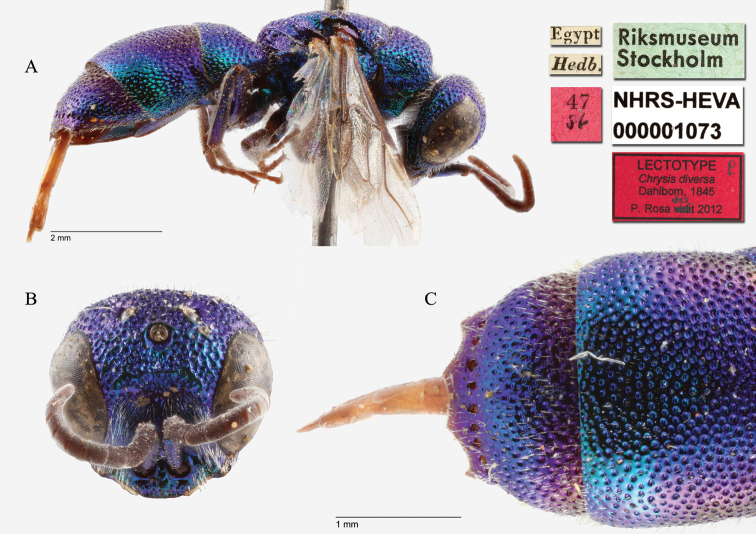
*Chrysis diversa* Dahlbom, 1845, lectotype (photo HV). **A** Habitus, lateral view **B** head, frontal view **C** second and third metasomal tergites, dorsal view.

(here designated) ♀: [Egypt] [Hedb.] [47 *86*] <red label> [Riksmuseum Stockholm] <green label> <red label> [Paralectotypus *Chrysis diversa* ♀ *Dahlbom 1845 des. by Bohart* P. Rosa vidit 2010] <red label> [*Chrysis palliditarsis* Spinola P. Rosa det. 2010] [NHRS-HEVA000001073] (Plate 5).

#### Paralectotype 1 ♀.

[Egypt] [Hedb.] [48 *86*] <red label> [Riksmuseum Stockholm] <green label> <red label> [Paralectotypus *Chrysis diversa* ♀ *Dahlbom 1845 des. by Bohart* P. Rosa vidit 2010] <red label> [*Chrysis palliditarsis* Spinola P. Rosa det. 2010] [NHRS-HEVA000001074].

#### Remarks.

Dahlbom (1845: 13) described *Chrysis diversa* without any information on the type-series. Later Dahlbom (1854: 226) listed that he examined only two specimens: “*Habitat in Aegypto, a D. Hedenborg detecta. Specimina duo e Museo R. Acad. Scient. Stockholm. communicavit D. Boheman*.” In the collection three female specimens are found. They bear red labels with the numbers 47, 48, 49 and they were all collected by Hedenborg in Egypt. Two specimens are equal and belong to the species *Chrysis palliditarsis* Spinola, 1838; whereas the third specimen, although with similar colouration and habitus, is different and belongs to the species *Chrysis viridissima* Klug, 1845. The latter specimen is not part of the original type-series and cannot be considered as syntype. The other two specimens, found in the collection with catalogue numbers 47 (NHRS-HEVA000001073) and 48 (NHRS-HEVA000001074) can be considered as syntypes. Bohart (in Kimsey and Bohart 1991: 446) designated the lectotype of *Chrysis diversa* and placed it in synonymy with *Chrysis palliditarsis*. Unfortunately, Bohart selected the specimen not syntypic and not belonging to *Chrysis palliditarsis* (n° 49), but the specimen belonging to *Chrysis viridissima*. It bears the labels: [Egypt] [Hedb.] [49 *86*] <red label> [Riksmuseum Stockholm] <green label> [*Chrysis diversa* ♀ *Dahlbom Lectotype R.M. Bohart*] <red label> [NHRS-HEVA000001131]. This specimen must be excluded from the type-series because the anal margin is quite different from the anal margin of *Chrysis diversa* as found in the original description: “*Abdominis segmenti 3:tii series ante-apicalis e punctis modicis non confluentibus constituta; dentes apicales breves obtusi. Corpus 2 ½ lin. long*”. All three specimens share the same shape of the pit row of the third tergite, but only two specimens have apical teeth short and more or less obtuse and their body lenght is “2 ½ lin.”. The female of *Chrysis viridissima* has different anal teeth: the median ones are rounded and the lateral ones are spiniform; moreover it is longer than the other two specimens. More differences are found between the two species (e.g. the length of the malar space (Plate 5B)) but without relation to the original description. According to the ICZN (Art. 74.2) if it is demonstrated that a specimen designated as a lectotype was not a syntype, it loses its lectotype status.

We here designate one of the two female syntypes as the lectotype of *Chrysis diversa* Dahlbom, 1845 to fix the synonym *Chrysis diversa* Dahlbom = *Chrysis palliditarsis* Spinola. If we would consider Bohart’s lectotype designation as valid, then the synonym *Chrysis diversa* Dahlbom = *Chrysis viridissima* Dahlbom would generate confusion, since *Chrysis diversa* has the priority over *Chrysis viridissima*, which is currently in prevailing use.

#### Current status.

*Chrysis palliditarsis* Spinola, 1838 (synonymised by Kimsey and Bohart 1991: 446).

### Chrysis elvira

Taxon classificationAnimaliaHymenopteraChrysididae

Balthasar, 1957

Plate 6

Chrysis elvira : Balthasar 1957: 151.

#### Type locality.

Afghanistan: “*Umgebung von Sarekanda (4100m) in Badakschan-gebirge (28.VII.1953)*”.

#### Holotype ♀.

[J. Klapperich Sarekanda, 4100m 28.7.53, Gebirge Badakschan NO – Afghanistan] [*Chrysis elvira* n.sp. Balth. ♀ Holotypus] <red label handwritten by Balthasar] [NHRS-HEVA000001080].

**Plate 6.**
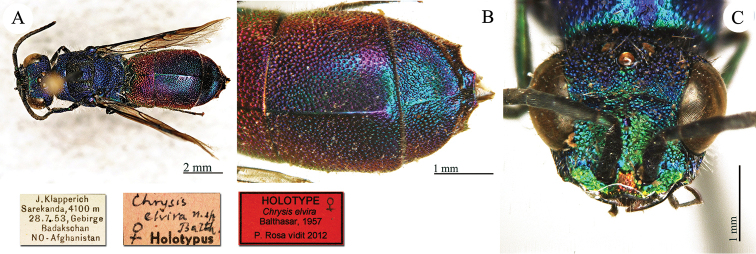
*Chrysis elvira* Balthasar, 1957, holotype. **A** Habitus, dorsal view **B** metasoma, dorsal view **C** head, frontal view.

#### Remarks.

One paratype found in the Linsenmaier Collection at the NMLS.

#### Current status.

*Chrysis elvira* Balthasar, 1957.

### Chrysis equestris

Taxon classificationAnimaliaHymenopteraChrysididae

Dahlbom, 1854

Plate 7

Chrysis equestris : Dahlbom 1854: 307.

#### Type locality.

unknown.

#### Holotype ♀.

[Mus. Payk.] [Type] [Typus] <red label> [374 *58*] <red label> [Naturhistoriska Riksmuseet Stockholm Loan no 993/98] <green label> [NHRS-HEVA000000008].

**Plate 7.**
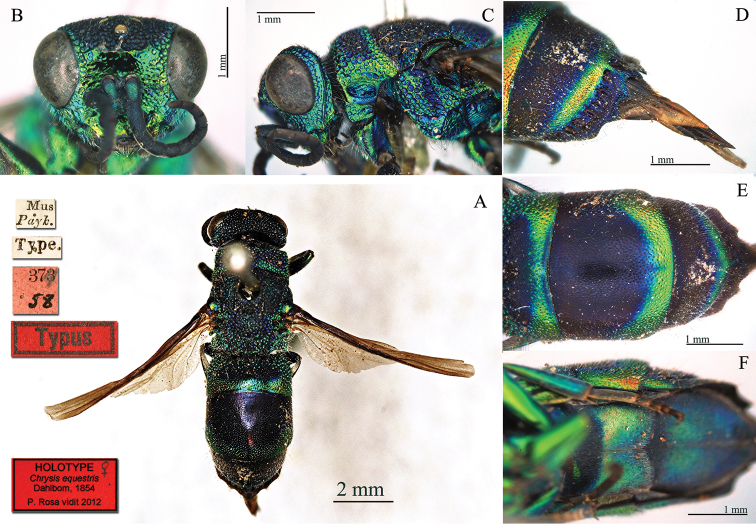
*Chrysis equestris* Dahlbom, 1854, holotype. **A** Habitus, dorsal view **B** head, frontal view **C** head and mesosoma, lateral view **D** third metasomal tergite, dorso-lateral view **E** metasoma, dorsal view **F** metasomal sternites, ventral view.

#### Remarks.

Dahlbom (1845: 11) described *Chrysis zetterstedti* based on a type series including male and female from Sweden and another specimen from Norway. Later Dahlbom (1854: 307) described the female as a separate species ‘*Specimen unicum e Collectione Paykulli Mus. R. Acad. Scient. Stockholm, communicavit D. Boheman*’. This specimen is both syntype of *Chrysis zetterstedti* Dahlbom, 1845 and holotype of *Chrysis equestris* Dahlbom, 1854. Both types of *Chrysis zetterstedti* and *Chrysis equestris* have been examined by Linsenmaier (1959: 163); the other two males (not syntypes) of *Chrysis zetterstedti* listed by Dahlbom (1854: 305) are housed in MZLU (Paukkunen et al. 2014).

#### Current status.

*Chrysis equestris* Dahlbom, 1854.

### Chrysis erythromelas

Taxon classificationAnimaliaHymenopteraChrysididae

Dahlbom, 1845

Plate 8

Chrysis erythromelas : Dahlbom 1845: 11.

#### Type locality.

unknown [not Italy, Sicily].

#### Holotype ♀.

[Mus. Payk.] [Type] [NHRS-HEVA000001081].

**Plate 8.**
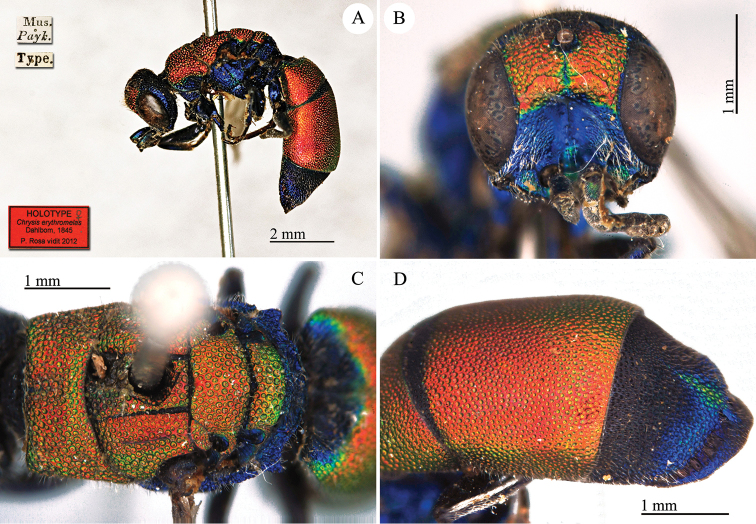
*Chrysis erythromelas* Dahlbom, 1845, holotype. **A** Habitus, lateral view **B** head, frontal view **C** mesosoma, dorsal view **D** second and third metasomal tergites, dorso-lateral view.

#### Remarks.

Dahlbom (1845: 11 [not 1854: 155]) described *Chrysis erythromelas* on a single specimen without any type locality, as written later more clearly by Dahlbom himself (1854: 155): “*Specimen e Collectione Pajkulliana Musei Reg. Acad. Scient. Stockholm. communicavit D. Boheman, patria non indicata*”. In the NHRS collection there are two specimens with the same label [Mus. Payk.] and belonging to the same species; but only one is labelled as [Type] and we consider it as the holotype. It is damaged after an old dermestid attack; it lacks the right flagellum, both right fore- and hindwing, left hindwing and right hindleg.

For a long time, *Chrysis erythromelas* has been considered as a variety of *Chrysis viridula* Linnaeus, 1761 by the most important authors (Mocsáry 1889: 444; Dalla Torre 1892: 108; Trautmann 1927: 165; Berland and Bernard 1938: 107). Kimsey and Bohart (1991: 424) synonymised it with *Chrysis integra* Fabricius, 1787. Without following the Principle of Priority, Linsenmaier (1951: 101) considered *Chrysis erythromelas* as a variety of *Chrysis cylindrica* Eversmann, 1857. Later Linsenmaier (1959: 132) placed *Chrysis erythromelas* in relation with Chrysis integra ssp. sicula Abeille de Perrin, 1877, but he was not sure about the correct relationship: “*Der Name* erythromelas *Dahlbom 1845 bezieht sich auf diese Spezies, doch kann ich nicht beurteilen, ob er als Synonym zu* integra *Nominatform aufzufassen ist, oder ob er an Stelle von ssp.* sicula *zu treten hätte (er wurde nach einem* ♀ *ohne Patria aufgestellt, auch ohne sichere Geschlechts-Bestimmung)*”. Finally Linsenmaier (1997a: 277) synonymised *Chrysis sicula* with *Chrysis ornata* Smith, 1851; but this synonym is in error, since *Chrysis ornata* is described from England and it is related to *Chrysis viridula* Linnaeus s. str.. *Chrysis integra* and related forms are distributed only in the Mediterranean area. The name *Chrysis erythromelas* was even used to identify other species belonging to the *Chrysis viridula* group. For example Invrea (1920: 417; 1921: 344) identified the females of *Chrysis pulcherrima* Lepeletier, 1806 as Chrysis bidentata var. erythromelas. The examination of the holotype confirms that *Chrysis erythromelas* is the first available name for the species named *Chrysis sicula* Abeille de Perrin, 1877 or Chrysis integra ssp. ornata Smith, 1851 *sensu* Linsenmaier (1997a) and widely distributed in northern Africa (see the material housed in the Linsenmaier collection) and in Sicily. The species is easily identifiable from *Chrysis integra* Fabricius by the deep and long frontal sulcus elongated between the fore ocellus and the facial scapal basin, halving the transversal frontal carina (TFC); punctation on metasoma with shining intervals between the punctures, with smaller dots between the larger punctures; last tergite with pit row deeply elongated (Plate 8).

#### Current status.

*Chrysis erythromelas* Dahlbom, 1845, **status revived.**

### Chrysis imperialis

Taxon classificationAnimaliaHymenopteraChrysididae

Dahlbom, 1845

Chrysis imperialis : Dahlbom 1845: 11.

#### Type locality.

Algeria.

#### Holotype ♂.

[Paykull] [Algier] [NHRS-HEVA000001089].

#### Remarks.

*Chrysis imperialis* Dahlbom, 1845 *nec* Westwood, 1842 is unavailable and the oldest available name from among its synonyms is *Chrysis tricolor* Lucas, 1849. However the validity of this species is not clear and currently it is considered a north African subspecies of *Chrysis semicincta* Lepeletier, 1806.

#### Current status.

Chrysis semicinta ssp. tricolor Lucas, 1849 (Linsenmaier 1959: 124).

### Chrysis jugum

Taxon classificationAnimaliaHymenopteraChrysididae

Dahlbom, 1850

Plate 9

Chrysis Jugum : Dahlbom 1850: 136.

#### Type locality.

South Africa: “*Natal*”.

#### Holotype ♀.

[Caffraria] [J. Wahlb.] [*jugum*] [269 *82*] <red label> [NHRS-HEVA000001090].

**Plate 9.**
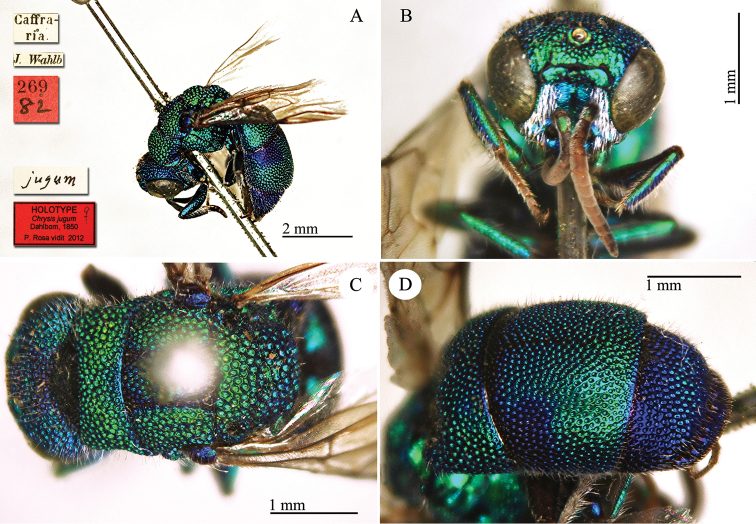
*Chrysis jugum* Dahlbom, 1850, holotype. **A** Habitus, lateral view **B** head, frontal view **C** head and mesosoma, dorsal view **D** metasoma, dorso-lateral view.

#### Current status.

*Chrysis jugum* Dahlbom, 1850.

### Chrysis klapperichi

Taxon classificationAnimaliaHymenopteraChrysididae

Balthasar, 1957

Chrysis klapperichi : Balthasar 1957: 148.

#### Type locality.

Afghanistan: “*Umgebung von Schau (2000m) im Kokscha-Tal in Badakschan-Gebirge (19.VII.1953)*”.

#### Holotype ♀.

[J. Klapperich Schau, 2000 m 19.7.53, Kokschatal, Badakschan NO – Afghanistan] [*Chrysis klapperichi* ♀ *n.sp. Balth.* Holotypus] [NHRS-HEVA000001092].

#### Remarks.

In Kimsey and Bohart (1991: 436) the type repository is reported as the NMPC.

#### Current status.

Chrysis martinella ssp. solox Semenov, 1954 (synonymised by Linsenmaier 1968: 74).

### Chrysis grohmanni ssp. krkiana

Taxon classificationAnimaliaHymenopteraChrysididae

Linsenmaier, 1959

Chrysis grohmanni ssp. krkiana : Linsenmaier 1959: 109.

#### Type locality.

Croatia: Krk island.

#### Paratype 1 ♂.

[*Insel Krk leg. Mader* Coll. Linsenmaier] [Chrysis ♂ grohmanni krkiana *Lins.* Linsenmaier det. *59*] [NHRS-HEVA000001093].

#### Paratype 1 ♀.

[*Insel Krk leg. Mader* Coll. Linsenmaier] [Chrysis ♀ grohmanni krkiana *Lins.* Linsenmaier det. *59*] [NHRS-HEVA000001094].

#### Remarks.

The two specimens do not bear the typical handwritten note ‚paratype‘ by Linsenmaier; but after the study of his collection in NMLS we can state that they are paratypes. Often Linsenmaier labelled only the holotype and the allotype, especially when describing subspecies with long series. These two specimens were donated by Linsenmaier and have the same handwritten locality and year of identification as the other specimens belonging to the type-series in the Linsenmaier collection. This subspecies is clearly separated from the nominal form (Rosa 2003: 307).

#### Current status.

Chrysis grohmanni ssp. krkiana Linsenmaier, 1959.

### Chrysis lateralis

Taxon classificationAnimaliaHymenopteraChrysididae

Dahlbom, 1845

Plate 10

Chrysis lateralis : Dahlbom 1845: 10.

#### Type locality.

Greece: Rhodes.

#### Syntype 1 ♀.

[Rhodus] [Hedenb.] [NHRS-HEVA000001095].

**Plate 10.**
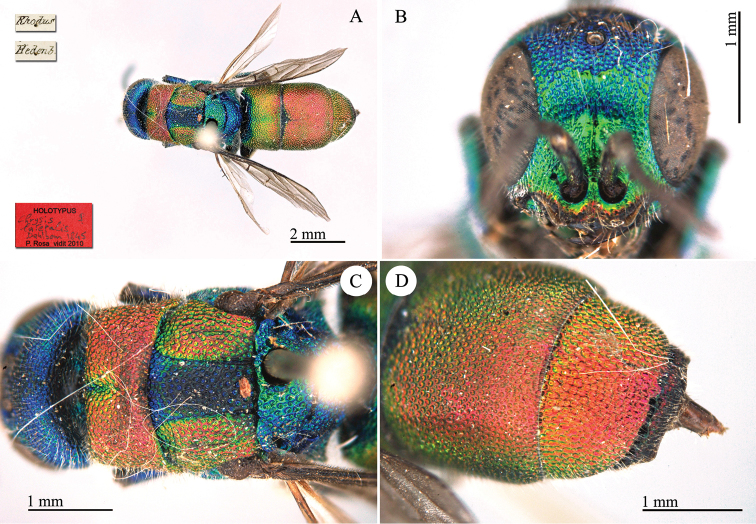
*Chrysis lateralis* Dahlbom, 1845, holotype. **A** Habitus, dorsal view **B** head, frontal view **C** mesosoma, dorsal view **D** second and third metasomal tergites, dorso-lateral view.

#### Remarks.

This species belongs to the *Chrysis elegans* group and is conspecific with *Chrysis separata* Trautmann, 1926. None of the most important authors (Dahlbom 1854; Mocsáry 1889; du Buysson (in André) 1891–1896; Trautmann 1927; Berland and Bernard 1938; Linsenmaier 1951, 1959, 1968) mentioned this species. Only Dalla Torre (1892: 74) and later Kimsey and Bohart (1991: 431) listed it as a valid species in the *comparata-scutellaris* group, without type examination. The female syntype has all the typical characteristics of *Chrysis separata*, species widespread from Zante (typical locality) to Middle East. A second syntype is housed in the Dahlbom collection in MZLU. We here propose *Chrysis separata* Trautmann, 1926, as a **new synonym** of *Chrysis lateralis* Dahlbom, 1845.

A similar case was found studying Dahlbom’s type of *Chrysis confluens* (Dahlbom, 1845). *Chrysis confluens* was described from Rhodes and belongs to the *Chrysis elegans* group. *Chrysis confluens* was synonymised by Dahlbom himself (1854: 159, var. h) with *Chrysis elegans* Lepeletier, 1806 and remained in synonymy with *Chrysis elegans* in all the most important works. However, nobody noticed that the description was perfectly matching the description of Chrysis elegans var. smaragdula Trautmann, 1926 *nec* Fabricius, 1775, also described from Rhodes. Linsenmaier (1959: 137) replaced the name *Chrysis elegans smaragdula* Trautmann with *Chrysis interrogata* Linsenmaier, 1959 without taking care of the possible synonymy with *Chrysis confluens* (Dahlbom). There is no doubt about Chrysis elegans var. smaragdula Trautmann, 1926 (currently Chrysis elegans ssp. interrogata Linsenmaier) as a **new synonym** of *Chrysis confluens* (Dahlbom, 1845), because *Chrysis confluens* is one of the most common species on the island and its peculiar colour is unique in this species group: “*Corpus æneo- aut subaurato-viride*” and “*Caput et thorax cyaneo- et viridi-variegata. Abdom. segmenti 3:tii series punctorum ante apicalis numerosorum orbiculatorum subconfluentium. Corpus 2 ½ lin. long.*”. This peculiar green or golden-green colouration is well emphasized by the name *smaragdula*, which in Latin means emerald green.

Both names *Chrysis separata* and *Chrysis interrogata* have been used mainly by Linsenmaier and a few other authors (i.e. Rosa 2005b; Strumia and Yildirim 2009), and according to the ICZN there is no reason for applying the Reversal of Precedence. The type of *Chrysis confluens* is housed in the Dahlbom collection in MZLU.

#### Current status.

*Chrysis lateralis* Dahlbom, 1845.

### Chrysis lucifera

Taxon classificationAnimaliaHymenopteraChrysididae

Bohart, 1982

Chrysis lucifera : Bohart (in Bohart & Kimsey) 1982: 123.

#### Type locality.

U.S.A. (holotype from Tanbark Flat, Los Angeles Co., California; paratypes: 11 ♂♂ and 41 ♀♀ from California, Idaho, Nevada, Oregon, Utah, Washington, Wyoming).

#### Paratype 1 ♂.

[Mt. Diablo, Cal. V-12-1937] [R.M. Bohart Colr] [Paratype Chrysis ♂ lucifera R.M. Bohart] <red label> [NHRS-HEVA000001097].

#### Paratype 1♀.

[Hopland Grade Lake Co. Cal. V-19-1961] [S.M. Fidel Collector] [Paratype Chrysis ♀ lucifera R.M. Bohart] <red label> [NHRS-HEVA000001098].

#### Remarks.

The holotype is deposited at the BME.

#### Current status.

*Chrysis lucifera* Bohart, 1982.

### Chrysis manicata

Taxon classificationAnimaliaHymenopteraChrysididae

Dahlbom, 1854

Plate 11

Chrysis manicata : Dahlbom 1854: 276.

#### Type locality.

Greece: Rhodes.

#### Syntype 1 ♂.

[Rhodus] [Hedenb.] [det. W. Trautmann] [*Tetrachrysis pallidicornis var. chloris Mocsáry*] <handritten by Trautmann> [NHRS-HEVA000001099].

#### Syntype 1 ♂.

[Rhodus] [Hedenb.] [det. W. Trautmann] [NHRS-HEVA000001100].

**Plate 11.**
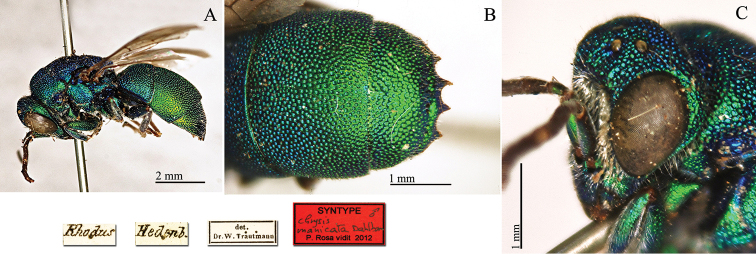
*Chrysis manicata* Dahlbom, 1854, syntype. **A** Habitus, lateral view **B** second and third metasomal tergites, dorsal view **C** head, lateral view.

#### Remarks.

In MNHU there are two other syntypes, male and female, labelled: [Rhodus, Mai, Loew S.] [*manicata Dahlb*. ♂] [Bischoff det.] [Syntypus *Chrysis manicata* ♂ *Dahlbom* P. Rosa vidit 2010] <in red>.

#### Current status.

*Chrysis manicata* Dahlbom, 1854.

### Chrysis modica

Taxon classificationAnimaliaHymenopteraChrysididae

Dahlbom, 1850

Plate 12

Chrysis modica : Dahlbom 1850: 140.

#### Type locality.

South Africa: “*Natal*”.

#### Lectotype ♀.

[Caffraria] [J. Wahlb.] [Typus] <red label> [*Chrysis modica Dahlb*.] [270 *82*] <red label> [*Chrysis modica Dahlbom Lectotype* ♀ *R.M. Bohart*] <red label> [NHRS-HEVA000001111].

**Plate 12.**
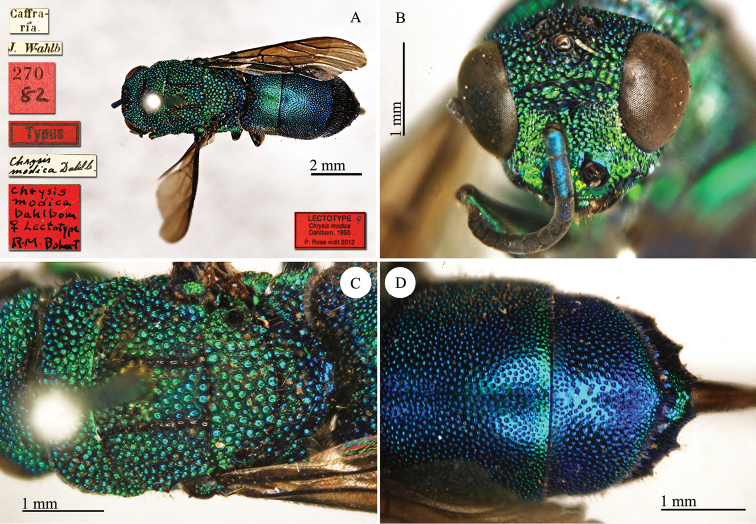
*Chrysis modica* Dahlbom, 1850, lectotype. **A** Habitus, dorsal view **B** head, frontal view **C** mesosoma, dorsal view **D** second and third metasomal tergites, dorsal view.

#### Remarks.

Dahlbom (1845: 14) described *Chrysis mediocris* on a single specimen from Guinea, received by Westermann and currently housed in his collection at the MZLU. Later Dahlbom (1850: 140) described the same species under a new name, *Chrysis modica*, adding one specimen from Port Natal collected by J. Wahlberg and deposited at the NHRS, and another specimen from Promontorium Bonae Spei [= Cape of Good Hope] found in Spinola’s collection (MRSN). In his last work Dahlbom (1854: 326) gave a detailed description in Latin of *Chrysis modica*, and described a new European species with the name *Chrysis mediocris* Dahlbom, 1854. The latter is a junior homonym of *Chrysis mediocris* Dahlbom, 1845 (currently *Chrysis subsinuata* Marquet, 1879). The first available name for *Chrysis modica* Dahlbom, 1850 is therefore *Chrysis mediocris* Dahlbom, 1845. Bohart (in Kimsey and Bohart 1991: 437) designated the lectotype.

#### Current status.

*Chrysis mediocris* Dahlbom, 1845 (synonymised by Kimsey and Bohart 1991: 437).

### Chrysis nisseri

Taxon classificationAnimaliaHymenopteraChrysididae

Dahlbom, 1845

Plate 13

Chrysis Nisseri : Dahlbom 1845: 14.

#### Type locality.

Columbia: “*Remedios*”.

#### Holotype ♀.

[Remedios] [Nisser] [Type] [NHRS-HEVA000001113].

**Plate 13.**
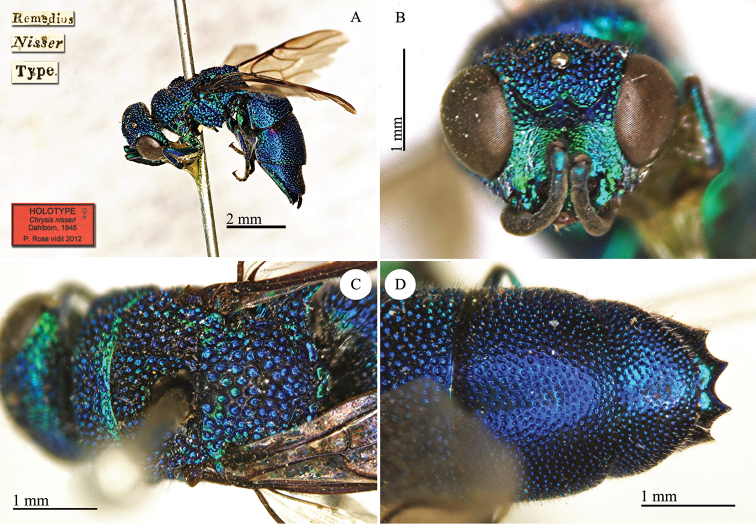
*Chrysis nisseri* Dahlbom, 1845, holoype. **A** Habitus, lateral view **B** head, frontal view **C** mesosoma, dorsal view **D** metasoma, dorsal view.

#### Remarks.

Dahlbom (1845) described *Chrysis nisseri* based on a female collected by Nisser at Remedios. Kimsey and Bohart (1991: 443) examined a male holotype in MZLU. This specimen was not located at the MZLU.

#### Current status.

*Chrysis nisseri* Dahlbom, 1845.

### Chrysis obsoleta

Taxon classificationAnimaliaHymenopteraChrysididae

Dahlbom, 1845

Plate 14

Chrysis obsoleta : Dahlbom 1845: 8.

#### Type locality.

unknown.

#### Holotype ♂.

[Mus. Payk.] [*Chrysis ignita var. obsoleta Dahlb. Dispos. 1845*] [NHRS-HEVA000000856].

**Plate 14.**
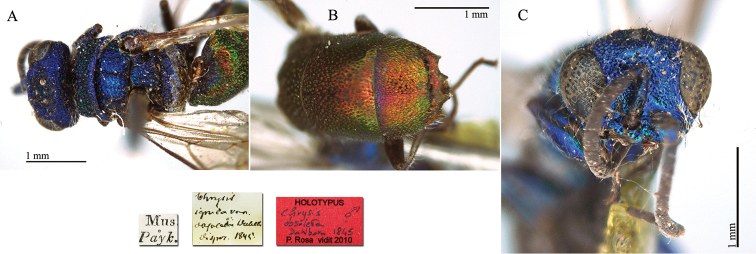
*Chrysis obsoleta* Dahlbom, 1845, holotype. **A** head and mesosoma, dorsal view **B** second and third metasomal tergites, dorsal view **C** head, frontal view.

#### Remarks.

The specimen is badly conserved. The metasoma was broken and glued using large quantity of glue, which now includes also part of the legs. All the European authors and Kimsey and Bohart (1991: 420) considered this small and slender specimen as synonym of *Chrysis ignita* (Linnaeus). The specimen clearly belongs to another species, probably *Chrysis angustula* Schenck, 1856. Villu Soon (pers. comm.) confirmed that it possibly belongs to *Chrysis angustula* but perhaps even *Chrysis solida* Haupt, 1956. Since the name *Chrysis obsoleta* Dahlbom has the priority on almost all the other names in the *ignita* group, we suggest considering it as a **nomen oblitum**, to maintain the prevailing usage of the names within this complicated species-group (Art. 23.9 of the Code).

#### Current status.

*Chrysis ignita* (Linnaeus, 1758) (synonymised by Mocsáry 1889: 488).

### Chrysis prominula

Taxon classificationAnimaliaHymenopteraChrysididae

Dahlbom, 1845

Plate 15

Chrysis prominula : Dahlbom 1845: 14.

#### Type locality.

unknown.

#### Holotype ♀.

[Mus. Payk.] [*prominula*] [NHRS-HEVA000001114].

**Plate 15.**
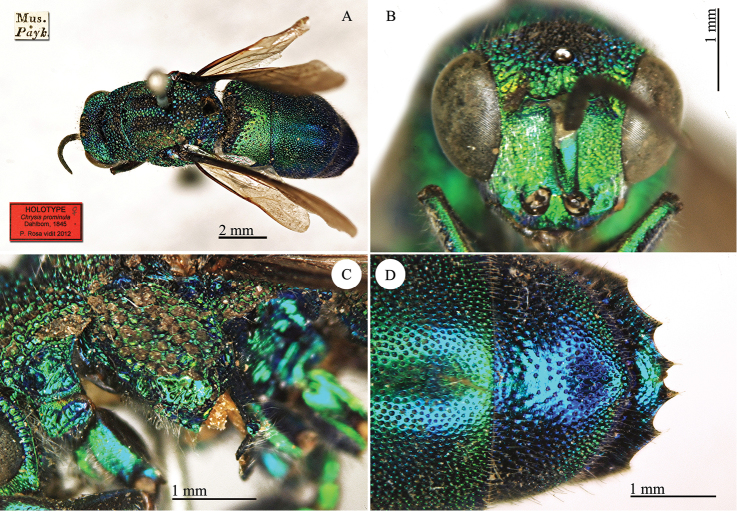
*Chrysis prominula* Dahlbom, 1845, holotype. **A** Habitus, dorsal view **B** head, frontal view **C** mesopleuron, lateral view **D** third metasomal tergite, dorsal view.

#### Remarks.

The type is partially damaged after an old dermestid attack. It lacks the right antenna, the left mid- and hindlegs, partially the right forewing and part of the metanotum.

#### Current status.

*Chrysis prominula* Dahlbom, 1845.

### Chrysis purpureifrons ssp. helleniensis

Taxon classificationAnimaliaHymenopteraChrysididae

Linsenmaier, 1968

Chrysis (Chrysogona) purpureifrons ssp. helleniensis : Linsenmaier 1968: 48.

#### Type locality.

Greece (holotype from Athen; allotype from Corinth; paratype localities not listed).

#### Paratypes 2 ♂♂ and 1 ♀.

[Graecia, Pelop. *18 km Südlich Tripolis 15.V.62* leg. Linsenmaier] [*Paratypen* Chrysis L. purpureifrons helleniensis *Lins.* Linsenmaier det. *63*] <handwritten in red> [NHRS-HEVA000001087].

#### Remarks.

The holotype is housed in the Linsenmaier Collection at NMLS.

#### Current status.

Chrysura purpureifrons ssp. helleniensis (Linsenmaier, 1968) (transferred by Kimsey and Bohart 1991: 494).

### Chrysis pyrrhina

Taxon classificationAnimaliaHymenopteraChrysididae

Dahlbom, 1845

Plate 16

Chrysis pyrrhina : Dahlbom 1845: 9.

#### Type locality.

unknown.

#### Holotype ♂.

[Mus. Payk.] [Type] [*pyrrhina Dahlbom 143*] [NHRS-HEVA000001115].

**Plate 16.**
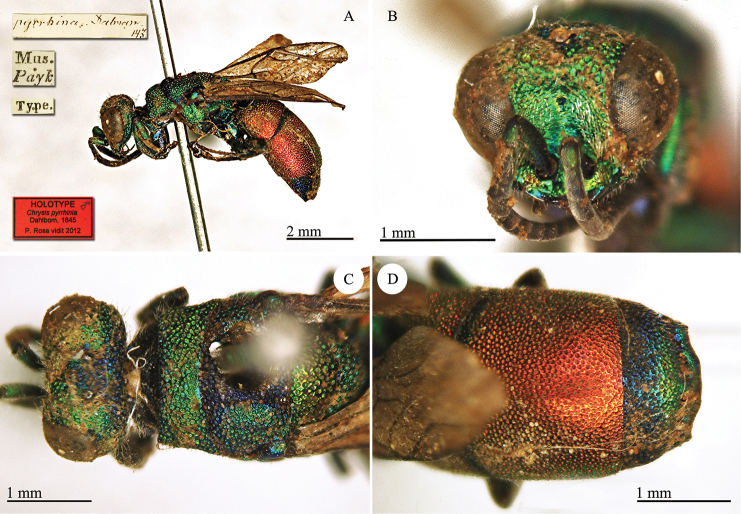
*Chrysis pyrrhina* Dahlbom, 1845, holotype. **A** Habitus, lateral view **B** head, frontal view **C** hed and mesosoma, dorsal view **D** metasoma, dorsal view.

#### Remarks.

The species was described with the name “*Chrysis pyrrhinia* Dalm. ♂ Mus. *Paykull*” and emendated in the same work (Dahlbom 1845: *corrigenda* at pag. 21). The type locality reported by Kimsey and Bohart (1991: 454 “Yugoslavia, Dalmatia”) is in error. Possibly they confused Dalm. [= Dalman] with Dalmatia. The type locality is unknown, as confirmed in Dahlbom (1854: 259): “Chrysis pyrrhina *Dalman Mus.* Paykulli; *teste D.* Boheman, *qui specimen unicum, patria non notata, e Museo R. Acad. Scient. Stockholm. amice communicavit*”.

Very likely Paykull received the male (described as *pyrrhina*) and the female (described as *erythromelas*) together, from the same locality, probably in north Africa. They both belong to the same species, *Chrysis erythromelas* Dahlbom, 1845, even if the male shows some peculiar characteristics which are not found in other northern African or Sicilian specimens: short pronotum, lateral angles on T-III more acute. The metasoma is entirely reddish, but this unusual colour was found also in other specimens in the Linsenmaier collection.

After Linsenmaier (1959) the name *Chrysis pyrrhina* was used to identify a common Mediterranean species (Mingo 1994; Mingo and Gayubo 1985, 1986a, 1986b; Mingo et al. 1988, 1990; Rosa 2004, 2005a, 2005b; Strumia 1995, 1996, 2005, 2007a, 2007b; Strumia and Pagliano 2010; Strumia et al. 2010). The type of *Chrysis pyrrhina* does not match Linsenmaier’s interpretation of the species and a new name must be given to this species.

The first available name from among its synonyms is *Chrysis serena* Radoszkowski, 1891. The type of *Chrysis serena* was checked and it is currently housed in the Radoszkowski collection in ISEA-PAS (Rosa et al. 2015). Linsenmaier (1968: 82) considered *Chrysis serena* as a subspecies of *Chrysis pyrrhina*, with coarser and denser punctation on the metasoma, with micro-punctated intervals between the punctures and mesosoma greener in colour. The distribution given by Linsenmaier for *Chrysis serena* is: Persia, S Russia, Palestine, Syria, Asia Minor and Manchuria. It is well known that in the Euro-Asiatic chrysidids, patterns in punctation have a gradient, becoming coarser from west to east. Similarly many common *Chrysis* are greener in the eastern area of their distribution in Europe. *Chrysis serena* simply represents the eastern form with coarser punctation.

#### Current status.

*Chrysis erythromelas* Dahlbom, 1845.

### Chrysis rufiventris

Taxon classificationAnimaliaHymenopteraChrysididae

Dahlbom, 1854

Plate 17

Chrysis rufiventris : Dahlbom 1854: 119.

#### Type locality.

unknown.

#### Holotype ♂.

[Mus. Payk.] [Type] [NHRS-HEVA000001116].

**Plate 17.**
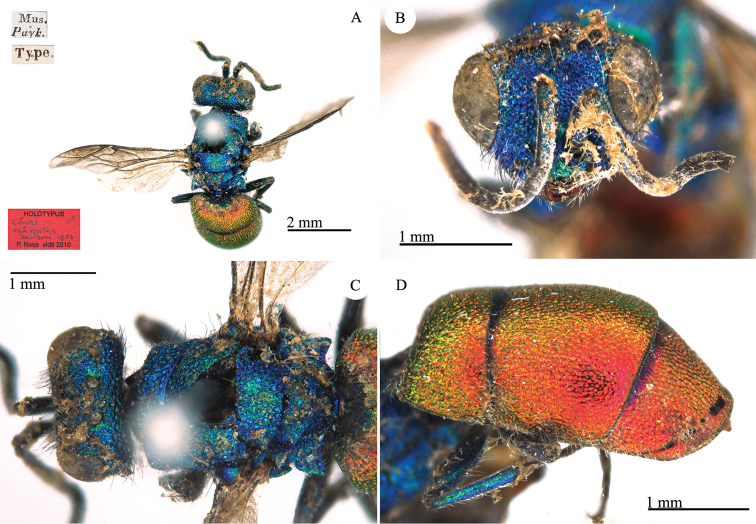
*Chrysis rufiventris* Dahlbom, 1854, holotype. **A** Habitus, dorsal view **B** head, frontal view **C** head and mesosoma, dorsal view **D** metasoma, lateral view.

#### Current status.

*Chrysura rufiventris* (Dahlbom, 1854) (tranferred by Kimsey and Bohart 1991: 495).

### Chrysis schoenherri

Taxon classificationAnimaliaHymenopteraChrysididae

Dahlbom, 1845

Plate 18

Chrysis Scönherri : Dahlbom 1845: 10.

#### Type locality.

South Africa.

#### Holotype ♀.

[Caffraria] [J. Wahlb.] [Typus] <red label> [*Chrysis schoenherri Dahlb*.] [271 *82*] <red label> [NHRS-HEVA000001118].

**Plate 18.**
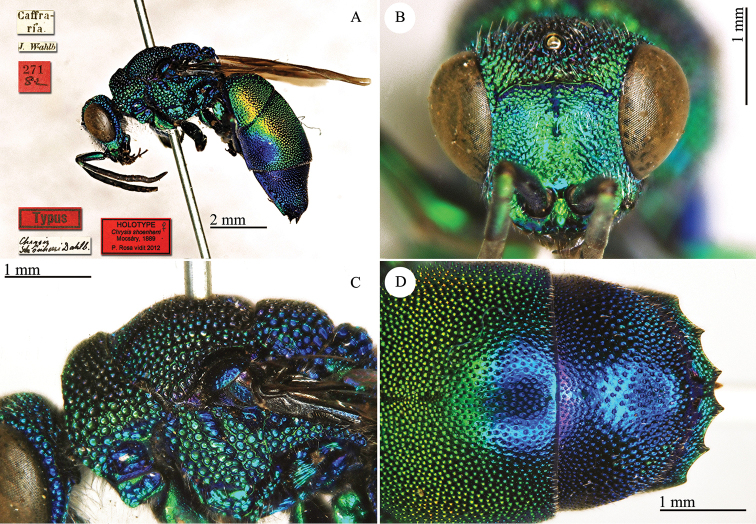
*Chrysis schoenherri* Dahlbom, 1845, holotype. **A** Habitus, lateral view **B** head, frontal view **C** mesosoma, lateral view **D** third metasomal tergite, lateral view.

#### Remarks.

The type lacks the left forewing. The emendated name *Chrysis schönherri* was introduced by Dahlbom (1850: 139). Carl Johan Schönherr was a Swedish entomologist born in Stockholm from a German family.

#### Current status.

*Chrysis schoenherri* Dahlbom, 1845.

### Chrysis scintillans

Taxon classificationAnimaliaHymenopteraChrysididae

Valkeila, 1971

Plate 19

Chrysis scintillans : Valkeila 1971: 85.

#### Type locality.

Finland: “*Vanaja*”.

#### Paratype 1 ♀.

[*Jmt. Undersåker* [unreadeable] *16.7.48* [*Valliste 1000m C.B. Gaunitz*] [Chrysis L. mediata ssp. fenniensis *Lins.* Linsenmaier det. *59*] [Chrysis scintillans *n.sp.* det. E. Valkeila – 69] [Typus] <red label> [NHRS-HEVA000001117].

**Plate 19.**
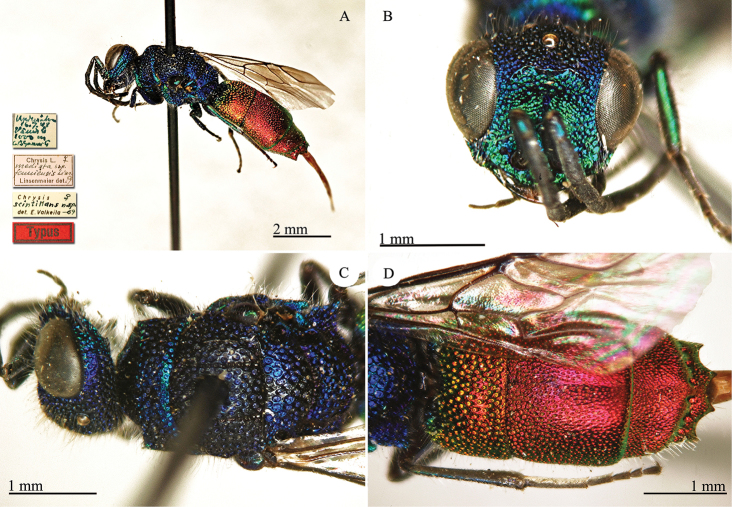
*Chrysis scintillans* Valkeila, 1971, paratype. **A** Habitus, lateral view **B** head, frontal view **C** head and mesosoma, dorsal view **D** metasoma, dorsal view.

#### Remarks.

The paratype lacks both right wings. The correct name for the locality of this paratype is found in the original description. The holotype is deposited at the MZH. *Chrysis scintillans* was described based on seventy-six specimens from Finland, Russia and Sweden. Paukkunen et al. (2014: 41) synonymised it with *Chrysis solida* Haupt, 1956, and found that the paratypes belong to different species: *Chrysis schencki* Linsenmaier, 1968 (36 exx.), *Chrysis solida* Haupt, 1956 (33 exx.), *Chrysis ignita* group (2 exx.), *Chrysis impressa* Schenck, 1856 (2 exx.), *Chrysis angustula* Schenck, 1856 (1 ex.) and *Chrysis subcoriacea* Linsenmaier, 1959 (1 ex.). The paratype preserved in NHRS belongs to *Chrysis schencki* (V. Soon and J. Paukkunen, in litt.). Valkeila considered the punctation of the terga as a more important character in species identification rather than other characteristics, such as the width of the ovipositor.

#### Current status.

*Chrysis solida* Haupt, 1956 (synonymised by Paukkunen et al. 2014: 41).

### Chrysis sinuata

Taxon classificationAnimaliaHymenopteraChrysididae

Dahlbom, 1845

Plate 20

Chrysis sinuata : Dahlbom 1845: 12.

#### Type locality.

South Africa: “*Capitis Bonae Spei*”.

#### Syntype 1 ♀.

[Cap. B. Spei] [Mus. Payk.] [NHRS-HEVA000001119].

**Plate 20.**
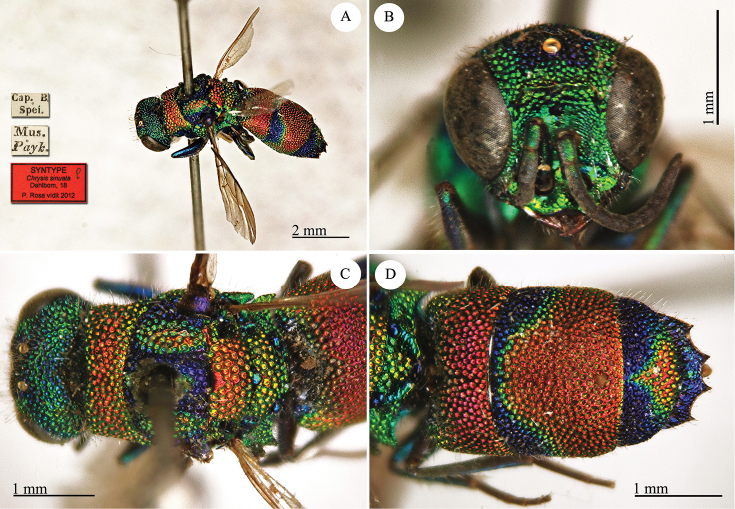
*Chrysis sinuata* Dahlbom, 1845, syntype. Syntype. **A** Habitus, dorso-lateral view **B** head, frontal view **C** head and mesosoma, dorsal view **D** metasoma, dorsal view.

#### Remarks.

The name *Chrysis sinuata* Dahlbom, 1845, *nec* Brullé, 1833 is not available and was replaced by Mocsáry (1889) with the name *Chrysis poecila*. See the other notes under *Chrysis sinuosa* Dahlbom.

#### Current status.

*Chrysis poecila* Mocsáry, 1889, a replacement name for *Chrysis sinuata* Dahlbom, 1845 *nec* Brullé, 1833.

### Chrysis sinuosa

Taxon classificationAnimaliaHymenopteraChrysididae

Dahlbom, 1854

Plate 21

Chrysis sinuosa : Dahlbom 1854: 153.

#### Type locality.

South Africa: “*Capitis Bonae Spei*”.

#### Holotype ♂.

[Cap. B. Spei] [Mus. Payk.] [Type] [*sinuosa Dahlbom 84*] [NHRS-HEVA000001120].

**Plate 21.**
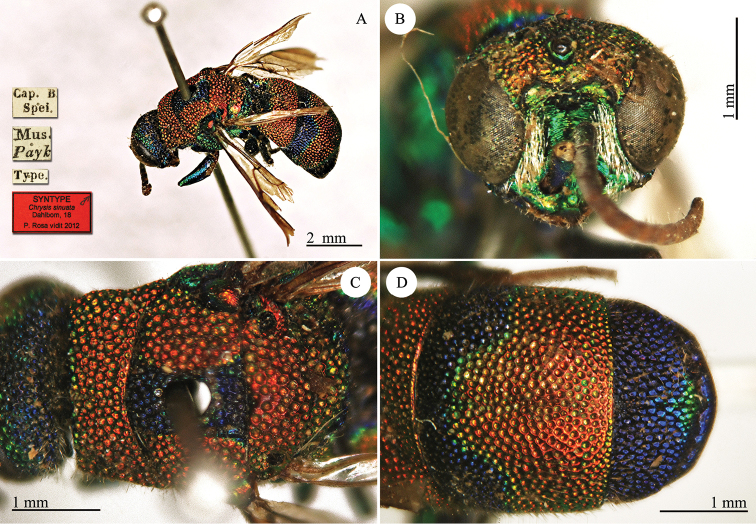
*Chrysis sinuosa* Dahlbom, 1854, holotype. Syntype. **A** Habitus, dorso-lateral view **B** head, frontal view **C** mesosoma, dorsal view **D** second and third metasomal tergites, dorsal view.

#### Remarks.

The type of *Chrysis sinuosa* lacks the right flagellum and the right foreleg. Dahlbom (1845: 11-12) described *Chrysis sinuata* based on two syntypes, a male and a female, with the same colour “*Divis IV. Thorax variegatus. Abdomen cyaneo-. viridi- et aureo-fasciatum*”. He described the female with four teeth on the anal margin (“*Subdivis. 2. Abdominis segmentum 3:tium apice 4-dentatum*”) (Plate 20D) and the male without teeth on the anal margin, but with a simple undulation (“*Subdiv. 3. Abdominis segmentum 3:tium apice undulatum*”) (Plate 21D). These two specimens clearly belong to two different species-groups. Later, Dahlbom (1854: 153) recognised the male as belonging to a different species and described it with the name *Chrysis sinuosa*. He left the female under the name *Chrysis sinuata* Dahlbom, 1845, without noticing that this name was already used by Brullé (1833).

Mocsáry (1889: 296), without type examination, considered *Chrysis sinuosa* Dahlbom, 1845 and *Chrysis sinuata* Dahlbom, 1854 (“*ex parte, solum* ♂” [the male only]) as synonyms of *Chrysis bellula* Guérin-Méneville, 1842. Mocsáry (1889: 440) also replaced the name *Chrysis sinuata* Dahlbom, 1845, *nec* Brullé, 1833 (“*ex parte, solum* ♀”) with *Chrysis poecila* Mocsáry, 1889. *Chrysis bellula* is now considered endemic to Madagascar, absent from South Africa (Azevedo et al. 2010: 858). Since Mocsáry (1889: 428) did not examine Dahlbom’s types, he described again *Chrysis sinuata* Dahlbom, 1845 as a new species from South Africa with the name *Chrysis eximia* Mocsáry, 1889. We examined the type of *Chrysis eximia*, which is deposited at the NHMW.

In this case, the replacement name *Chrysis poecila* Mocsáry has priority over *Chrysis eximia* Mocsáry and therefore we propose the new synonym *Chrysis eximia* Mocsáry, 1889 = *Chrysis poecila* Mocsáry, 1889. Edney (1952: 423) followed Mocsáry in the interpretation of Chrysis (Holochrysis) bellula Brullé, but without reporting any differences between the sexes. He also described *Chrysis ceres* Edney, 1952, which resulted synonym of *Chrysis sinuosa* Dahlbom. Kimsey and Bohart (1991: 463), without the examination of Dahlbom’s types, synonymised *Chrysis sinuata* Dahlbom, *Chrysis poecila* Mocsáry and *Chrysis ceres* Edney with *Chrysis sinuosa* and used *eximia* Mocsáry, 1889 as the valid name. Madl and Rosa (2012) followed the interpretation given by Kimsey and Bohart (1991).

According to the types, the two valid species and their synonymies are:

1. *Chrysis poecila* Mocsáry, 1889 replacement name for *Chrysis sinuata* Dahlbom, 1845 *nec* Brullé, 1833 (synonyms: *Chrysis eximia* Mocsáry, 1889; *Chrysis westwoodi* Mocsáry, 1912) (*Chrysis splendidula-senegalensis* group);
2. *Chrysis sinuosa* Dahlbom, 1845 (synonym: *Chrysis ceres* Edney, 1954) (*Chrysis capitalis* group).

#### Current status.

*Chrysis sinuosa* Dahlbom, 1854.

### Chrysis soror

Taxon classificationAnimaliaHymenopteraChrysididae

Dahlbom, 1854

Plate 22

Chrysis soror : Dahlbom 1854: 240.

#### Type locality.

Greece: “*Habitat in insula Rhodo, a D. Hedenborg detecta; Mus. D. Loew*”.

#### Lectotype

(here designated) ♂: [Rhodus] [Hedenb.] [det. W. Trautmann] [*Tetrachrysis abbreviaticornis Buyss.* ??] <handritten by Trautmann> [NHRS-HEVA000001121].

**Plate 22.**
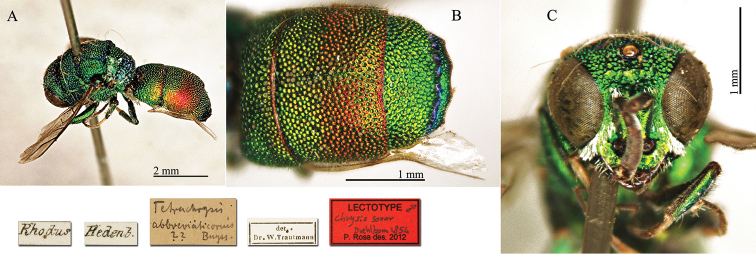
*Chrysis soror* Dahlbom, 1855, lectotype. **A** Habitus, lateral view **B** second and third metasomal tergites, dorsal view **C** head, frontal view.

#### Remarks.

Dahlbom (1854) described *Chrysis soror* based on more male specimens collected at Rhodes by Hedenborg and Loew. Kimsey and Bohart (1991: 464) listed the holotype in MNHU, but we could not find it with the help of the curator Frank Koch; Loew’s Chrysididae are not conserved in MNHU, as well as in BMNH or NHRS. Since it is possible that one or more syntypes could be found in another collection, we select the male specimen housed at the NHRS as lectotype, which matches perfectly the current interpretation of the species.

The lectotype is partially damaged; it lacks the left flagellum, tarsi of the right midleg and the left hindleg except for the coxa. The metasoma is glued to the mesosoma.

#### Current status.

*Chrysis soror* Dahlbom, 1854.

### Chrysis tenuimediata

Taxon classificationAnimaliaHymenopteraChrysididae

Linsenmaier, 1968

Chrysis (Papuachrysis) tenuimediata : Linsenmaier 1968: 53.

#### Type locality.

Burma: “*N.O. Burma, Kambaiti, 2000m*”.

#### Holotype ♀.

[N. E. Burma Kambaiti; 2000 m 23/4.1934 Malaise] [Riksmuseum Stockholm] <green label> [♀ *Type* Chrysis L. Papuachrysis tenuimediata *Lins.* Linsenmaier det. *64*] <handwritten in red> [Chrysis L. subgen. Adscitis det. Linsenmaier 19*94*] [NHRS-HEVA000001126].

#### Remarks.

Linsenmaier (1997a: 284) disagreed with the placement of *Chrysis tenuimediata* proposed by Kimsey and Bohart (1991) and described the subgenus Chrysis (Adscitis) based on *Chrysis tenuimediata*.

#### Current status.

*Primeuchroeus tenuimediatus* (Linsenmaier, 1968) (transferred by Kimsey and Bohart 1991: 543).

### Chrysis violacuna

Taxon classificationAnimaliaHymenopteraChrysididae

Bohart, 1982

Chrysis violacuna : Bohart (in Bohart & Kimsey) 1982: 134.

#### Type locality.

U.S.A. (holotype, 59 ♂♂ and 56 ♀♀ paratypes from Utah).

#### Paratype 1♂.

[UTAH Rich Co. S.W. Shore Bear Lake Reared, FD Parker] [16605F Rearing No.] [Paratype Chrysis violacuna ♂ R.M. Bohart] <red label> <pinned with cocoon> [NHRS-HEVA000000858].

#### Paratype 1♀.

[UTAH Rich Co. S.W. Shore Bear Lake Reared, FD Parker] [16674C Rearing No.] [Paratype Chrysis violacuna ♀ R.M. Bohart] <red label> <pinned with cocoon> [NHRS-HEVA000000859].

#### Remarks.

The holotype is deposited at the BME.

#### Current status.

*Chrysis violacuna* Bohart, 1982.

### Chrysis wahlbergi

Taxon classificationAnimaliaHymenopteraChrysididae

Dahlbom, 1845

Plate 23

Chrysis Wahlbergi : Dahlbom 1845: 14.

#### Type locality.

South Africa: “*Natal*”.

#### Lectotype ♂.

[Caffraria] [J. Wahlb.] [272 *82*] <red label> [*wahlbergi*] [*Lectotype Chrysis wahlbergi* ♂ *Dahlbom R.M. Bohart*] <red label> [NHRS-HEVA000001127].

**Plate 23.**
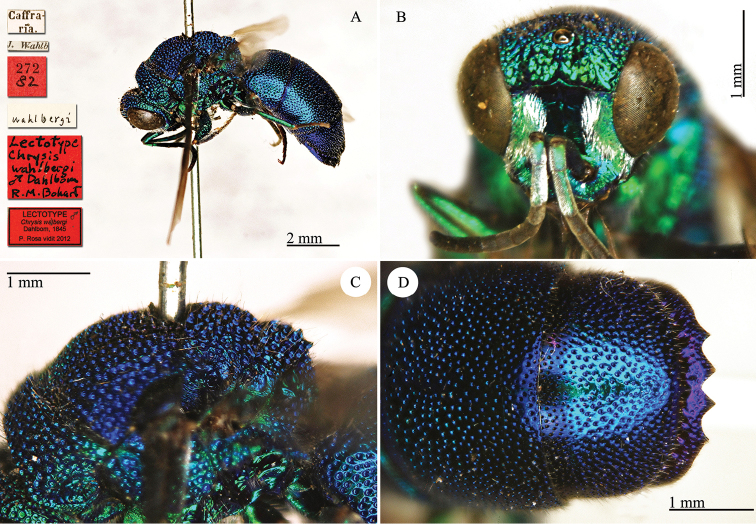
*Chrysis wahlbergi* Dahlbom, 1845, lectotype. **A** Habitus, lateral view **B** head, frontal view **C** mesosoma, lateral view **D** second and third metasomal tergites, dorsal view.

#### Paralectoype ♀.

[Caffraria] [J. Wahlb.] [273 *82*] <red label> [*wahlbergi*] [NHRS-HEVA000001128].

#### Remarks.

Lectotype designation by Bohart (in Kimsey and Bohart 1991: 478).

#### Current status.

*Chrysis wahlbergi* Dahlbom, 1845.

### Chrysura candens

Taxon classificationAnimaliaHymenopteraChrysididae

Dahlbom, 1845

Chrysura candens : Dahlbom 1845: 7.

#### Type locality.

Greece: Rhodes.

#### Holotype ♀.

[Mus. Payk.] [NHRS-HEVA000001066].

#### Remarks.

*Chrysura candens* Dahlbom is a secondary junior homonym of *Chrysis candens* Germar, 1817. Dalla Torre (1892: 49, 58) placed *Chrysis candens* in synonym of *Chrysis candens* Klug (!) Germar, 1817 (partim) and *Chrysis elegans* Lepeletier (partim). The type is surely related to large species (*3 ½ lin. long.*), not comparable with *Chrysis candens* Germar. The type is partially damaged; it lacks the left fore wing, femura, tibiae and tarsi of left mid- and hindlegs, and tarsi of the left foreleg.

#### Current status.

*Chrysis elegans* Lepeletier, 1806 (synonymized by Mocsáry 1889: 301).

### Chrysura foveata

Taxon classificationAnimaliaHymenopteraChrysididae

Dahlbom, 1845

Plate 24

Chrysura foveata : Dahlbom 1845: 6.

#### Type locality.

Egypt.

#### Syntype ♀.

[Egypt] [Hedb.] [NHRS-HEVA000001083].

#### Syntype ♂.

[Egypt] [Hedb.] [NHRS-HEVA000001084].

**Plate 24.**
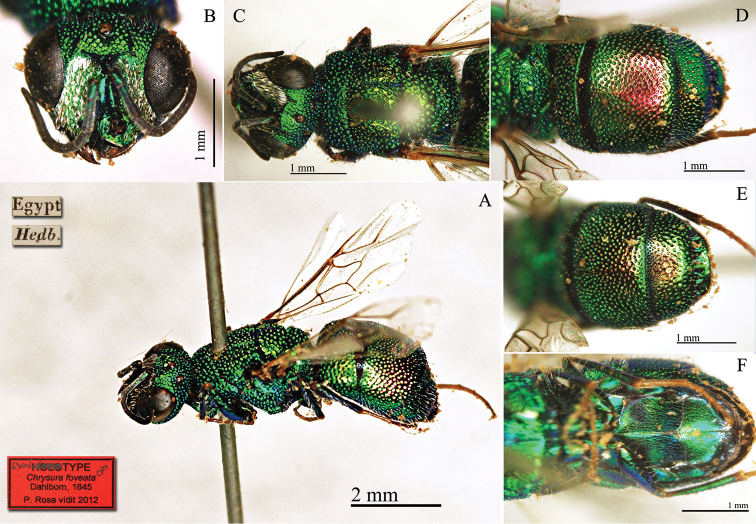
*Chrysura foveata* Dahlbom, 1845, syntype male. **A** Habitus, dorso-lateral view **B** head, frontal view **C** head and mesosoma, dorsal view **D** metasoma, dorsal view **E** third metasomal tergite, dorsal view **F** metasomal sternites, ventral view.

#### Remarks.

*Chrysura foveata* was described based on few specimens (*rar.*) considered as females, but in the collection one female and one male are found. Dahlbom (1854: 172) gave a subsequent description of the species, which is not very precise, especially with respect to the colour. Kimsey and Bohart (1991: 497) placed *Chrysura foveata* in synonym with *Chrysura trimaculata* (Förster, 1853), without type examination. This synonym is in error; *Chrysura foveata* was described from Egypt, whereas *Chrysura trimaculata* is a Euro-Sibiric species, not distributed in northern Africa and belonging to a different genus. *Chrysura foveata* belongs to the *Chrysis hydropica* group.

#### Current status.

*Chrysis foveata* (Dahlbom, 1845) (transferred by Mocsáry 1889: 292).

### Chrysura humboldti

Taxon classificationAnimaliaHymenopteraChrysididae

Dahlbom, 1845

Plate 25

Chrysura Humboldti : Dahlbom 1845: 6.

#### Type locality.

Greece: Rhodes.

#### Holotype ♂.

[Rhodus] [Hedb.] [Type] [NHRS-HEVA000001088].

**Plate 25.**
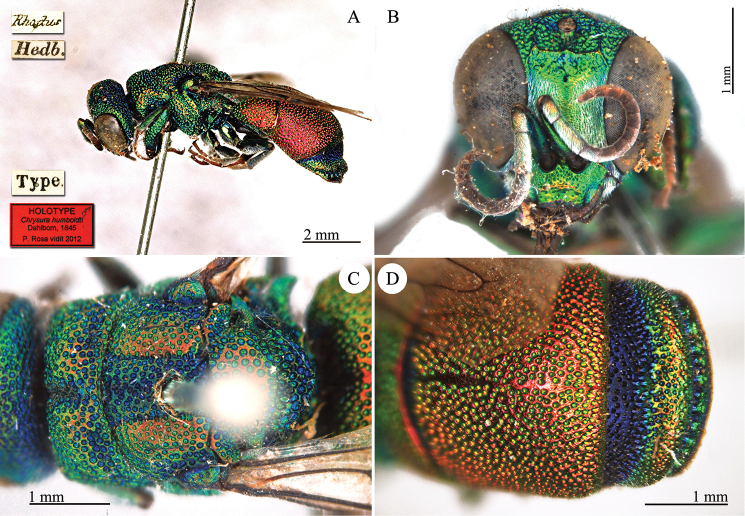
*Chrysura humboldti* Dahlbom, 1845, holotype. **A** Habitus, lateral view **B** head, frontal view **C** mesosoma, dorsal view **D** second and third metasomal tergites, dorsal view.

#### Current status.

*Pseudospinolia humboldti* Dahlbom, 1845 (transferred by Kimsey and Bohart 1991: 547).

### Chrysura sulcata

Taxon classificationAnimaliaHymenopteraChrysididae

Dahlbom, 1845

Plate 26

Chrysura sulcata : Dahlbom 1845: 7.

#### Type locality.

Greece: Rhodes.

#### Lectotype

(here designated) ♀: [Rhodus] [Hedb.] [Type] [NHRS-HEVA000001125].

**Plate 26.**
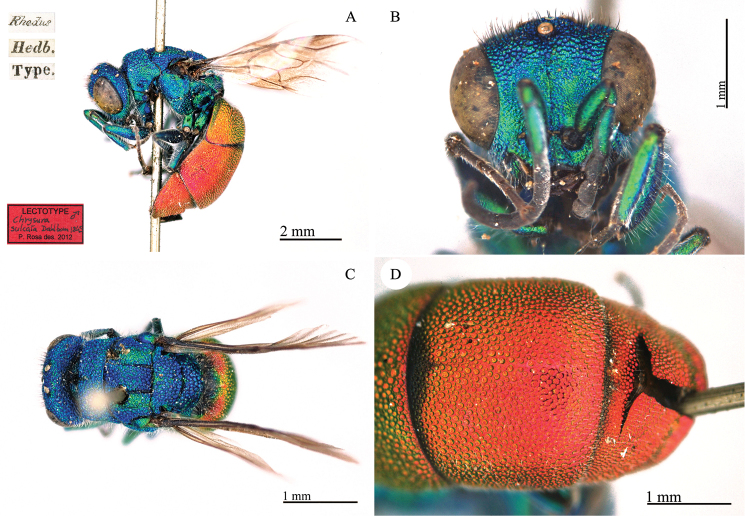
*Chrysura sulcata* Dahlbom, 1845, lectotype. **A** Habitus, lateral view **B** head, frontal view **C** habitus, dorsal view **D** second and third metasomal tergites, dorso-lateral view.

#### Notes.

Dahlbom (1845) described *Chrysura sulcata* based few specimens from Rhodes “*Chrysura sulcata nob. Rhodus rar. Hedenborg.*”. Since Dahlbom wrote “*rar.*” and not “*rariss.*” he examined at least two specimens. The original diagnosis (Dahlbom 1845) is quite different from the description given in 1854 and the current interpretation of the species. Dahlbom (1845) described *sulcata* as a species with red sternites “*Divis. 2. Abdominis dorsum totum aureum. Venter igneus*”, whereas the species today identified as *Chrysura sulcata* has blue or blue-green sternites, which is a useful characteristic to separate it from *Chrysura rufiventris* Dahlbom, 1854 in south Europe. Moreover, he described *Chrysura sulcata* with green mesosoma (“*Thorax viridis*”), whereas all the specimens studied have blue mesosoma, with green reflections on the lateral sides of the mesonotum.

In the general collection, under the name *Chrysura sulcata*, two specimens were found. These are a specimen of *Chrysura sulcata* and a second specimen (without head), which belongs to *Chrysis aestiva* Dahlbom, 1845, also described from Rhodes. It bears the same labels: [Rhodes] [Hedb.]. This specimen is obviously different, since it has two small teeth along the anal margin; but we noticed that the position of the ovipositor somehow hides the two small teeth. Perhaps it is possible that Dahlbom did not see these two small teeth and considered this specimen as a syntype. The latter has red sternites and green mesosoma.

Later Dahlbom (1854: 116), after the examination of a Sicilian specimen housed at the NHMW, gave a better and detailed description of the species, which was accepted by all the following authors and is currently recognised. The specimen examined at the NHMW is lost and was considered as a syntype by Kimsey and Bohart (1991). Since the original description is ambiguous and the species could be described from several specimens, in accordance with the ICZN (Art. 73) we hereby designate the lectotype of *Chrysura sulcata* on the male specimen bearing the label [Type] and characterised by the broken last tergite (Plate 26D). The designated lectotype matches the current interpretation of the species given by Dahlbom (1854) and Linsenmaier (1959).

#### Current status.

*Chrysura sulcata* Dahlbom, 1845.

### Cleptes fasciata

Taxon classificationAnimaliaHymenopteraChrysididae

Dalman, 1823

Plate 27

Cleptes fasciata : Dalman 1823: 90.

#### Type locality.

Brazil.

#### Lectotype ♀.

[*Brasilia Freyreiss*] [Schh.] <Schönherr> [Naturhistoriska Riksmuseet Stockholm Loan no 333/96] <green label> [Cleptidea ♀ fasciata *Dalm.* det. L. Móczár, 1996] [Lectotypus *Cleptes fasciata Dalman des. Móczár 996*] <red label> [NHRS-HEVA000001082].

**Plate 27.**
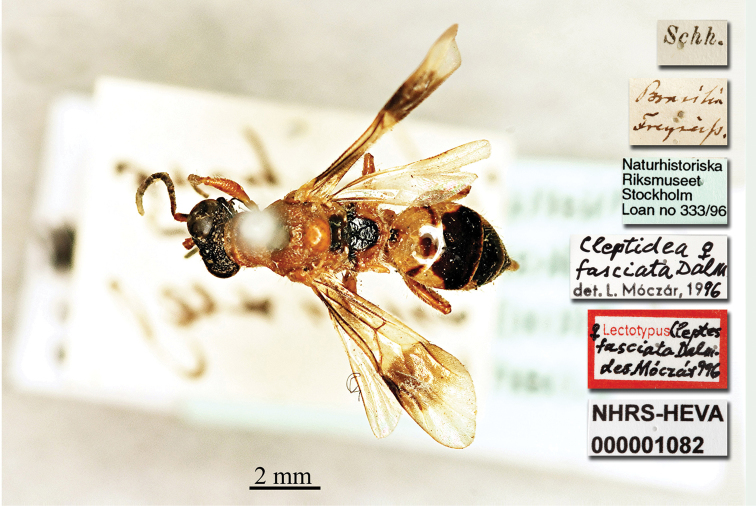
*Cleptes fasciata* Dalman, 1823, holotype. Habitus, dorsal view.

#### Remarks.

Lectotype designated by Móczár (1996: 136); a paralectotype is deposited at the HNHM.

#### Current status.

*Cleptidea fasciata* (Dalman, 1823) (transferred by Mocsáry 1904: 569).

### Cleptes sjostedti

Taxon classificationAnimaliaHymenopteraChrysididae

Hammer, 1950

Cleptes Sjöstedti : Hammer 1950: 2.

#### Type locality.

China: “*Provinz Kiansu, leg. Kolthoff, Oktober*”.

#### Holotype ♀.

[Provins Kiangsu] [China Kolthoff] [Type] <red label> [*Cleptes sjöstedti mihi* ♀ det. Hammer] <handwritten by Hammer> [NHRS-HEVA000001124].

#### Remarks.

Hammer described *Cleptes sjostedti* based on two females, a holotype and a paratype. Móczár (1998a: 341) searched for the holotype in NHRS, but the senior curator, Fredrik Ronquist, could not find it. Consequently Móczár (1998a), according to the ICZN (Art. 75), designated the neotype based on the paratype housed in Hammer’s collection in NHMW. The discovery of the original holotype automatically sets aside Móczár’s neotype designation (Art. 75.8, status of rediscovered former name-bearing types). Pictures of the holotype are provided by Rosa et al. (2014).

The correct spelling of the name should be *sjostedti* and not *sjoestedti* as reported by Móczár (1998a: 325) and Kimsey and Bohart (1991: 435) in the following case of *Chrysis sjostedti* Cameron. These two species were dedicated to Yngve Sjöstedt, professor and curator of the NHRS; according to the ICZN (Art. 32.5.2.1); only in case of a German name the correct writing would be *sjoestedti*.

#### Current status.

*Cleptes sjostedti* Hammer, 1950.

### Cymura splendida

Taxon classificationAnimaliaHymenopteraChrysididae

Dahlbom, 1845

Cymura splendida : Dahlbom 1845: 4.

#### Type locality.

Turkey: “*Bosfor*”.

#### Holotype ♂.

[Bosfor Hed. 32] [NHRS-HEVA000001122].

#### Current status.

*Hedychrum coelestinum* Spinola, 1838 (synonymised by Dahlbom 1854: 60).

### Hedychrum massaicum

Taxon classificationAnimaliaHymenopteraChrysididae

Cameron, 1910

Plate 28

Hedychrum massaicum : Cameron 1910: 299.

#### Type locality.

Tanzania: “*Kilimandjaro. 2^nd^ November*”.

#### Holotype ♂.

[Kilimandj. Sjöstedt] [*2* Nov] [Typus] [*Hedychrum massaicum*] <handwritten by Cameron> [173 *85*] <red label> [Riksmuseum Stockholm] <green label> [*Holotype* Hedychrum massaicum ♂ *Cameron* det L D French] <red label> [NHRS-HEVA000001101].

**Plate 28.**
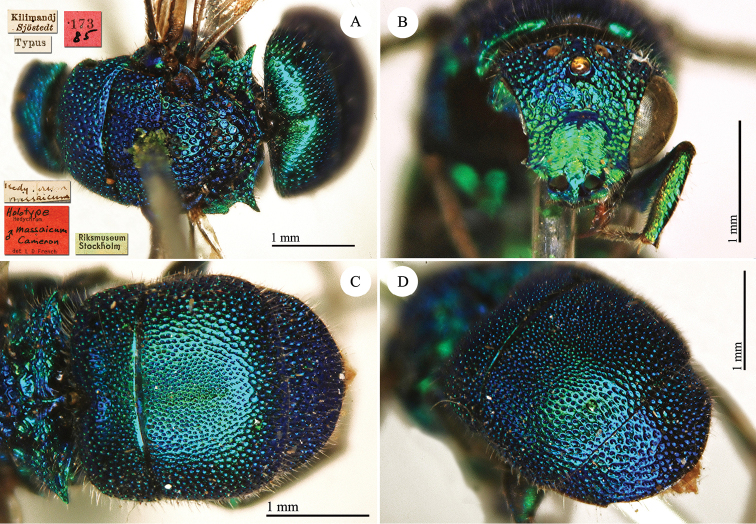
*Hedychrum massaicum* Cameron, 1910, holotype. **A** Habitus, dorsal view **B** head, frontal view **C** metasoma, dorsal view **D** second and third metasomal tergites, dorso-lateral view.

#### Remarks.

The type is badly damaged by dermestids. It lacks the antennae, the right part of the head, including mouthparts and occipitum and the right foreleg. Together with this type there are two other specimens ([NHRS-HEVA000001134] and [NHRS-HEVA000001135]) collected in the same locality by Sjöstedt and on Mount Meru, but to be excluded from the type-series because they were collected on different days. Cameron (1910) described *Hedychrum massaicum* based only on the specimen collected on the 2^nd^ of November. The other two specimens have been collected the 6^th^ of September and in January. French identified all of them as *Hedychrum massaicum* but one of the specimens [175 *85*] collected in January belongs to a different species. Bohart (Kimsey and Bohart 1991: 216) examined the “holotype” deposited at the MZLU, but this specimen was not found, and all the material collected by Sjöstedt is deposited at the NHRS.

#### Current status.

*Hedychrum massaicum* Cameron, 1910.

### Hexachrysis sjostedti

Taxon classificationAnimaliaHymenopteraChrysididae

Cameron, 1910

Hexachrysis Sjöstedti : Cameron 1910: 297.

#### Type locality.

Tanzania: “*Kilimandjaro: Kiboto, cultivated zone, 1,300-1,900 m. 7^th^ May*”.

#### Holotype ♀.

[Kilimandj. Sjöstedt] [Kibonoto 1300 – 1900 m] [Typus] [*Chrysis sjöstedti*] <handwritten by Cameron> [177 *85*] <red label> [Riksmuseum Stockholm] <green label> [Chrysis malachitica ♀ *Dahlbom* R.M. Bohart det.] [NHRS-HEVA000001123].

#### Remarks.

The type is seriously damaged. It lacks great parts of the head; a small part is still connected to the mesosoma and includes TFC, ocelli, right part of the face, including mandibles and part of the antenna; all the legs, sternites and internal tergites and sternites are lost. We compared this specimen with the type of *Chrysis malachitica* Dahlbom, 1854 (deposited at the ZMUC). Small differences exist in colour, punctation and shape of the pronotum, probably due to the distances between the two populations.

#### Current status.

*Chrysis malachitica* Dahlbom, 1854 (synonymised by Kimsey and Bohart 1991: 435).

### Holopyga amoenula

Taxon classificationAnimaliaHymenopteraChrysididae

Dahlbom, 1845

Plate 29

Holopyga amoenula : Dahlbom 1845: 4.

#### Type locality.

Greece: Rhodes.

#### Lectotype

(here designated) ♂: [Rhodus] [Hedb.] [NHRS-HEVA000001059].

**Plate 29.**
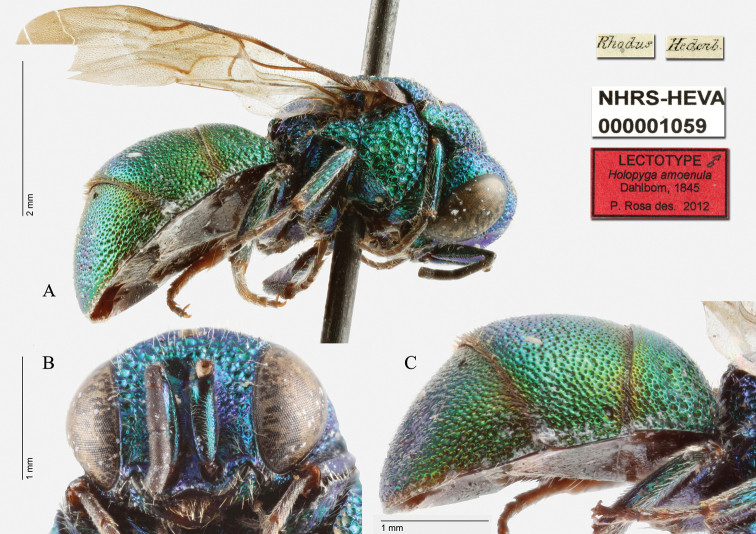
*Holopyga amoenula* Dahlbom, 1845, lectotype (photo HV). **A** Habitus, lateral view **B** head, frontal view **C** metasoma, lateral view.

#### Paralectotype 2 ♂♂.

[Rhodus] [Hedb.] [NHRS-HEVA000001060] and [NHRS-HEVA000001061].

#### Paralectotype 1 ♂.

[Rhodus] [Hedb.] [Naturhistoriska Riksmuseet Stockholm Loan no 188/96] [NHRS-HEVA000001062].

#### Paralectotype 1 ♂.

[Rhodus] [Hedb.] [det. dr. W. Trautmann] [Naturhistoriska Riksmuseet Stockholm Loan no 188/96] <green label> [NHRS-HEVA000000857].

#### Remarks.

*Holopyga amoenula* is the type species of *Holopyga* Dahlbom, 1845. In the general collection at the NHRS we found more similar specimens under the name “*Holopyga amoenula* Dahlbom” with the same labels: “Rhodus” and “Hedenborg”. They belong to different species. Dahlbom (1845: 4) wrote: ”*Holopyga amoenula* nob. ♂ Rhodus rar. *Hedenborg*”. It is not possible to know how many specimens he examined, but we guess few specimens (*rar.*), as written in the introduction. Later Dahlbom (1854: 53) wrote: “*Duo specimina ex Insula Rhodo vidi, unum a D. Hedenborg alterum a D. Loew lecta*.” The second specimen is not a type, because the material collected by Loew was not included in the original description.

The history of the name *Holopyga amoenula* is rather confused, since it was used by many authors to identify almost all the European species of *Holopyga*. The synthesis of this confused situation can be found in Kimsey and Bohart (1991: 225), where many species belonging to different species groups are placed in synonym with *Holopyga amoenula* (Rosa 2006: 136). More generally, the most common European species, currently known as *Holopyga generosa* (Förster, 1853) (= *ovata* Dahlbom, 1854) is found in synonym with *Holopyga amoenula* after Mocsáry’s monograph (1889: 127). The same taxonomical overview was proposed by Mingo (1994: 73, 204) whereas in the other most important monographs (i.e. Trautmann 1927: 50, and Berland and Bernard 1938: 42), *Holopyga amoenula* was considered as variety of *Holopyga gloriosa*. The name *gloriosa* Fabricius has been suppressed by the ICZN Commission (ICZN 1998, Opinion 1906) and the species previously identified with this name *sensu* Linsenmaier are related with a different species-group, which includes *Holopyga lucida* (Lepeletier), *Holopyga inflammata* (Förster), *Holopyga caucasica* Mocsáry, etc.

Only after Linsenmaier’s revision (1959) of the European species, *Holopyga amoenula* was correctly identified and recognized as a distinct, valid species endemic to Rhodes. The discussion on the name *amoenula* originates in Dahlbom’s monograph (1854: 53). Dahlbom considered *Holopyga amoenula* as variety (var. d) of the new described species *Holopyga ovata*, contrary to the Principle of Priority that was not yet applied at that time. Two subspecies of *Holopyga amoenula* are present in southern Europe: Holopyga amoenula ssp. oriensa Linsenmaier and ssp. occidenta Linsenmaier. The possibility that they could be valid species should be taken in consideration.

Since there are different specimens in the collection, and species collected by Hedenborg on Rhodes under the name *Holopyga amoenula*, we hereby designate as the lectotype the specimen which match the current interpretation of the species. It is pinned, in perfect condition and we dissected the genitalia, glued with the specimen (Plate 29).

#### Current status.

*Holopyga amoenula* Dahlbom, 1845.

### Holopyga dohrni

Taxon classificationAnimaliaHymenopteraChrysididae

Dahlbom, 1854

Plate 30

Holopyga dohrni : Dahlbom 1854: 48.

#### Type locality.

Cuba and U.S.A.: “*Habitat in Cuba Cel. Dohrn, in New York Cel. Kriechbaumer, qui mihi specimina amice donarunt.*”.

#### Paralectotype 1 ♂.

[*Cuba*] [*Dohrn*] [NHRS-HEVA000001075].

#### Paralectotype 1 ♂.

[*Cuba*] [*Dohrn*] [NHRS-HEVA000001076].

**Plate 30.**
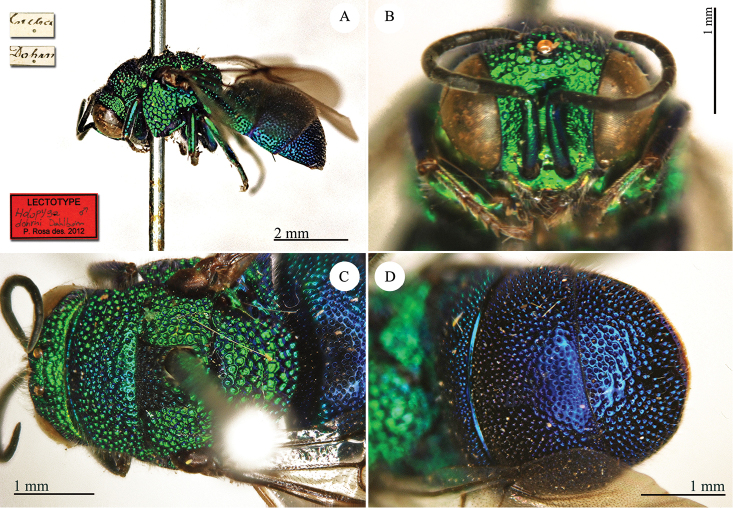
*Holopyga dohrni* Dahlbom, 1854, lectotype. **A** Habitus, lateral view **B** head, in frontal view **C** mesosoma, dorsal view **D** second and third metasomal tergites, dorsal view.

#### Remarks.

Dahlbom (1854) described *Holopyga dohrni* based on a type-series including specimens from Cuba, received from Dohrn, and New York, received from Kriechbaumer. Mocsáry (1889: 122) without type examination placed *Holopyga dohrni* Dahlbom in synonymy with of *Holopyga ventralis* (Say). This synonym was accepted by several authors (Dalla Torre 1892: 30, Bodenstein 1951: 720; Krombein 1979: 1225). Bohart and Kimsey (1982: 28) listed type “unknown” and placed *Holopyga dohrni* in synonymy with *Holopyga ventralis*, with restricted distribution to New York; later Bohart (in Kimsey and Bohart 1991: 236) examined the syntype collected in New York and considered it as a holotype. With this assumption (locality restricted to N.Y.) and term (holotype), Bohart (in Kimsey and Bohart 1991) explicitly indicated that he was selecting from the type series that particular specimen to serve as the name-bearing type (Art. 74.5). Therefore the syntype deposited at the MZLU must be considered as the lectotype.

The two Cuban paralectotypes collected by Dohrn are deposited at the NHRS and belong to a different species, probably to *Holopyga cyaniventris* (Cresson, 1865).

#### Current status.

*Holopyga ventralis* (Say, 1824).

### Omalus coriaceus

Taxon classificationAnimaliaHymenopteraChrysididae

Dahlbom, 1850

Plate 31

Omalus coriaceus : Dahlbom 1850: 135.

#### Type locality.

South Africa.

#### Holotype ♀.

[Caffraria] [J. Wahlb.] [Type] [NHRS-HEVA000000860].

**Plate 31.**
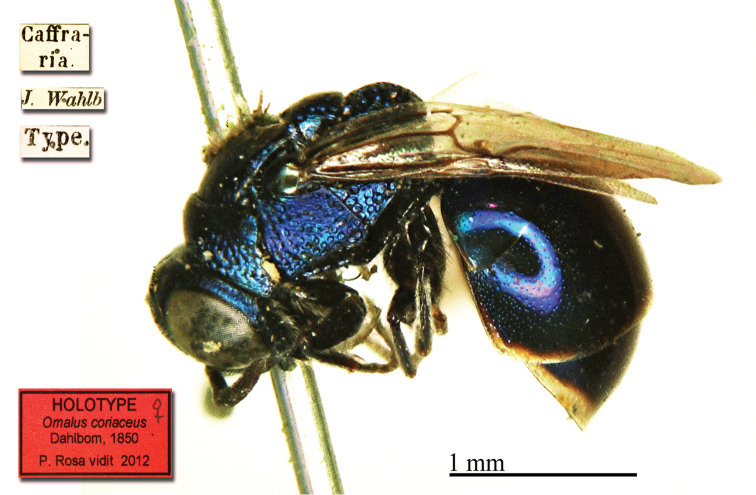
*Omalus coriaceus* Dahlbom, 1850, holotype. Habitus, lateral view.

#### Current status.

*Holophris coriaceus* (Dahlbom, 1850) (transferred by Mantero 1910: 548).

### Pentachrysis kibonotoensis

Taxon classificationAnimaliaHymenopteraChrysididae

Cameron, 1910

Pentachrysis kibonotoensis : Cameron 1910: 298.

#### Type locality.

Tanzania.

#### Holotype

♂ [not ♀]: [Kilimandj. Sjöstedt] [Kibonoto 1800-1900 m] [*Pentachrysis kibonotoensis ns* ♂] [Riksmuseum Stockholm] <green label> [176 *85*] <red label> [Praestochrysis spina ♂ *(Brullé)* R M Bohart det] [NHRS-HEVA000001091].

#### Remarks.

The type is seriously damaged by an old dermestid attack. It lacks the antennae (except scapus), the compound eyes, part of the scapal basin, tibia and tarsi of the left foreleg, both hindlegs and the sternites and internal urites. Also the first metasomal tergite is partially damaged.

#### Current status.

*Praestochrysis spina* (Brullé, 1846) (synonymised and transferred by Kimsey and Bohart 1991: 535).

### Platycelia ehrenbergi

Taxon classificationAnimaliaHymenopteraChrysididae

Dahlbom, 1845

Platycelia Ehrenbergi : Dahlbom 1845: 8.

#### Type locality.

Egypt.

#### Holotypus

**(?)** 1 ♀. [Egypt] [Hedb.] [NHRS-HEVA000001077- NHRS-HEVA000001079].

#### Remarks.

Dahlbom (1845: 8) described ”Platycelia Ehrenbergi *nob. Ægypt. rariss.* Hedenborg.”. The use of “*rariss*.” suggests that Dahlbom examined only one specimen. Confirmation is given by Dahlbom himself (1854: 220) “Habitat *in Aegypto, a D.* Hedenborg *detecta. Unicum specimen vidi, e Museo Reg. Acad. Scient. Stockholm. a D.* Boheman *communicatum.*”. In the collection three specimens belonging to the same species were located bearing the same labels. Boheman sent only one specimen of this series to Dahlbom, who described the species. Later the specimen was reintroduced in the original series and the label handwritten by Dahlbom was destroyed. Currently the holotype is “lost” within the series, and a neotype could be designated by the first revisor. We do not select a neotype, because all the three specimens correspond to the current interpretation of the species and therefore the neotype designation seems to be unnecessary.

A revision of the *Chrysis ehrenbergi* species-group is needed, because many subspecific names were proposed and their relation is not clear. Trautmann (1926: 7) described Cephalochrysis ehrenbergi var. vogti; Linsenmaier (1968: 106, 107) described three different subspecies: Chrysis (Platycelia) ehrenbergi ssp. vinaria, Chrysis ehrenbergi ssp. hylae, Chrysis ehrenbergi ssp. chrysodorsa (= *Chrysis ehrenbergi vogti* Trautmann). Linsenmaier (1968: 106) wrote that *Chrysis ehrenbergi* exists with different ecological and geographical forms: “ehrenbergi *Dhlb. existiert in, mindestens im* ♀ *Geschlecht, durch die Färbung deutlich getrennten, ökologischen und geographischen Formen. Die Nominatform scheint auf Ägypten beschränkl zu sein.* ♀ *grün, K und Th obern bronzefarben oder mit weniger intensiven kupfernen Reflexen, Abd oben rosa-kupfern.*”. However, some ecological or geographical forms could be valid species, as in the case of *Chrysis ignita* (Linnaeus).

Linsenmaier always considered *Platycelia* Dahlbom as a valid and well-characterized subgenus; Linsenmaier (1997a: 285) observed that Kimsey and Bohart (1991) elevated some subgenera to generic level (e.g. *Spintharina* Semenow), whereas other subgenera equally or even more characteristic (e.g. *Platycelia* Dahlbom, *Pyria* Lepeletier, etc.) were downgraded to species-group even if clearly separated from the heterogeneous genus *Chrysis* Linnaeus. The generic status and placement of *Platycelia* should be checked in the future, with the help of molecular analysis.

#### Current status.

*Chrysis ehrenbergi* (Dahlbom, 1845) (transferred by Dalla Torre 1892: 58).

### Stilbum hedenborgi

Taxon classificationAnimaliaHymenopteraChrysididae

Dahlbom, 1845

Stilbum Hedenborgi : Dahlbom 1845: 16.

#### Type locality.

Sudan: “*Bahr el Abiad*”.

#### Syntypes

2 ♀♀. [Bahr el Abiad] [Hedenborg] [NHRS-HEVA000001085] and [NHRS-HEVA000001086].

#### Current status.

*Chrysis stilboides* Spinola, 1838 (synonymised and transferred by Mocsáry 1889: 590).

### Stilbum wesmaeli

Taxon classificationAnimaliaHymenopteraChrysididae

Dahlbom, 1845

Plate 32

Stilbum Wesmaëli : Dahlbom 1845: 16.

#### Type locality.

Greece: Rhodes.

#### Holotype ♂.

[Rhodus] [Hedb.] [NHRS-HEVA000001129].

**Plate 32.**
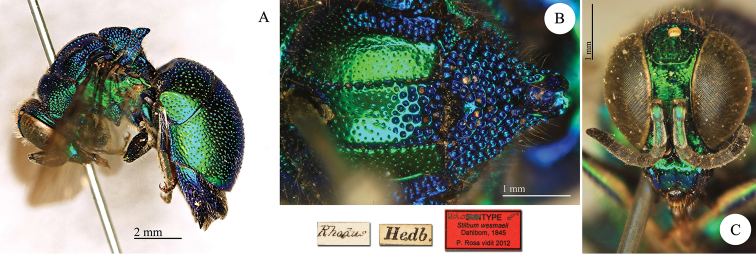
*Stilbum wesmaeli* Dahlbom, 1845, holotype. **A** Habitus, lateral view **B** mesonotum and metanotum, dorsal view **C** head, frontal view.

#### Remarks.

Dahlbom (1845) described *Stilbum wesmaeli* without any note on the type series. More information can be found in his monographical work (Dahlbom 1854: 359): “*Habitat in insula Rhodo; specimen unicum e Mus. Reg. Acad. Scient. Stockholm. communicavit Dom. Boheman*.”. Currently there are three specimens in the collection collected on Rhodes by Hedenborg. Only one has a different printed label [Hedb.] [NHRS-HEVA000001129] instead of [Hedenb.] [NHRS-HEVA000001137-1138]. Hedenborg visited Rhodes more than once, and these three specimens should have been collected in two different journeys. We consider as holotype the one with a different label (NHRS-HEVA000001129).

After Dahlbom (1854), all the most important authors considered *Stilbum wesmaeli* as synonym of *Stilbum cyanurum* (Forster, 1771) (Mocsáry 1889: 190; Dalla Torre 1892: 38; Bishoff 1913: 26; Trautmann 1927: 80; Linsenmaier 1951: 107). However, it was not even mentioned by Mader (1933) and Zimmermann (1937) in their revisions of the genus *Stilbum*. In his major revisions, Linsenmaier (1959: 181 and 1968: 123) used the name Stilbum calens ssp. subcalens Mader, 1933 (invalid name because described as *aberratio*) in place of *Stilbum wesmaeli* for the corresponding subspecies distributed in the Mediterranean basin (Dalmatia, Balcan Contries, Rhodes, Persia, southern Switzerland (Misox), southern France, Spanien, northern Africa (Linsenmaier 1959) and Lebanon (Linsenmaier 1968)).

In the last publications, Linsenmaier (1997a: 287, 1997b: 134, 1999: 254) used Stilbum calens ssp. wesmaeli Dahlbom as the oldest name for this species, synonymizing *Stilbum subcalens* and *Stilbum macedonicum* Trautmann, 1926 with Stilbum calens ssp. wesmaeli. Linsenmaier (1959) treated the invalid name *subcalens* as subspecies of *Stilbum calens*, and thus made this name available as species-group name (ICZN 1999, article 45.6.3.). As Linsenmaier was the first author to make the name available, he should be considered as the author of *Stilbum subcalens* (ICZN 1999, article 50.3.1.).

The type and the other specimens of *Stilbum wesmaeli* in NHRS are not related to *Stilbum calens* (Fabricius), but belong to a different population of *Stilbum cyanurum* (Forster) probably endemic to the island. Dahlbom (1845, 1854) descriptions are clear and this species is easily identifiable by the typical shape of the metanotal protrusion, which is deeply bilobed (“*postscutelli processus emarginatus*”). All the specimens from Rhodes show this special feature, and for this reason we consider this isolated population as a possible valid subspecies.

#### Current status.

Stilbum cyanurum ssp. wesmaeli (Dahlbom, 1845).

### Stilbum westermanni

Taxon classificationAnimaliaHymenopteraChrysididae

Dahlbom, 1845

Plate 33

Stilbum Westermanni : Dahlbom 1845: 16.

#### Type locality.

Greece: Rhodes.

#### Holotype ♂.

[Rhodus] [Hedb.] [NHRS-HEVA000001130].

**Plate 33.**
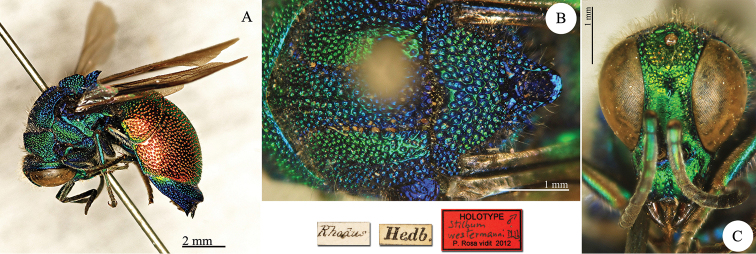
*Stilbum westermanni* Dahlbom, 1845, holotype. **A** Habitus, lateral view **B** mesonotum and metanotum, dorsal view **C** head, frontal view.

#### Remarks.

*Stilbum westermanni* is related to *Stilbum calens* Fabricius. Linsenmaier (1997a: 287, 1997b: 134, 1999: 254) confused the two species described by Dahlbom from Rhodes (*wesmaeli* and *westermanni*), and proposed the wrong combination Stilbum calens ssp. wesmaeli instead of Stilbum calens ssp. westermanni. According to Linsenmaier (1997b), this subspecies is more distributed along the coast of the Mediterranean basin.

#### Current status.

Stilbum calens ssp. westermanni Dahlbom, 1845.

### Missing types

During the revisional work in the general collection, the following types were not found, which should be deposited at the NHRS according to the literature.

#### Chrysis gloriosa

Taxon classificationAnimaliaHymenopteraChrysididae

Dahlbom, 1845

Chrysis gloriosa : Dahlbom 1845: 10, *nec* Fabricius, 1793

##### Type locality.

unknown.

##### Remarks.

Dahlbom (1845) based the description of *Chrysis gloriosa* on a specimen related to *Chrysis grohmanni* Dahlbom, 1854, as written by the same author (Dahlbom 1854: 271). Since the locality is unknown and many subspecies of *Chrysis grohmanni* have been described in the Mediterranean countries, it is impossible to comment this name.

#### Chrysis inaequalis

Taxon classificationAnimaliaHymenopteraChrysididae

Dahlbom, 1845

Plate 34

Chrysis inaequalis : Dahlbom 1845: 8.

##### Type locality.

Turkey: “*Bosfor*”.

##### Neotype

(here designated) ♂: [*Helvetia*] [*Roveredo 28.8.46*] [♂ Chrysis L. inaequalis D. det. Lins.] [NML_ENT GBIF_Chr 00038702] deposited at NMLS.

**Plate 34.**
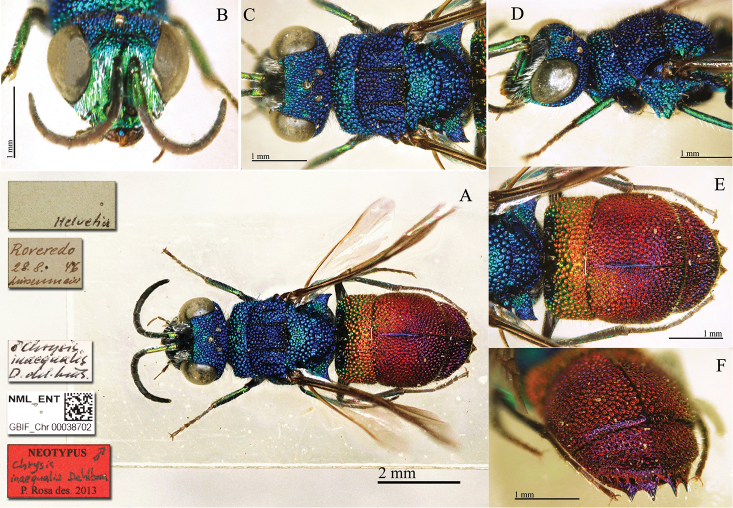
*Chrysis inaequalis* Dahlbom, neotype. **A** Habitus, dorsal view **B** head, frontal view **C** head and mesosoma, dorsal view **D** mesosoma, lateral view **E** metasoma, dorsal view **F** second and third metasomal tergites, dorso-lateral view.

##### Remarks.

*Chrysis inaequalis* is one of the most common species in Europe. It was described from Turkey (Bosfor), but the type is lost. In the general collection we could only find two females of *Chrysis inaequalis* collected at Rhodes by Hedenborg. According to Linsenmaier the “typical” *Chrysis inaequalis* is present only in central-, southern Europe and in northern Africa; in the rest of the distributional range, from Greece to central Asia, the subspecies *Chrysis inaequalis sapphirina* Semenov-Tian-Shanskij, 1912 is present. *Chrysis sapphirina* is the eastern form with green-coloured males and both sexes coarsely punctuated. Linsenmaier (1959) cited *Chrysis inaequalis* s. str. in North China and Manchuria, but later, in his collection, he identifed all the eastern specimens as Chrysis inaequalis ssp. sapphirina. Linsenmaier (1959) did not notice that the typical locality of *Chrysis inaequalis* correspond with the distribution given for Chrysis inaequalis ssp. sapphirina sensu auctorum.

For this reason a neotype designation of *Chrysis inaequalis* is needed. We could not find any other specimen from Bosphor (Istanbul and adjacent areas), but in Linsenmaier’s collection we found many specimens collected in western Turkey, both on the European and the Asiatic side. The closest localities are Edirne (on the European side) and Ayvalik (on the Asiatic side). Even if it is not required for a neotype designation, Ayvalik is a seaside town on the northwestern Aegean coast of Turkey, it is possible that Hedenborg visited this town moving from Rhodes or Egypt to Istanbul. In fact Hedenborg was the medical doctor of the Swedish Embassy at Istanbul, and not only a famous naturalist who published different papers on his journeys in Rhodes and Egypt.

However, since the name *Chrysis inaequalis* is in prevailing use for the identification of the western European specimens for the last 100 years, we prefer to designate a neotype based on one specimen collected in central Europe, rather than on a specimen collected nearby the typical locality. If we designate a neotype on an eastern Mediterranean species, the name *Chrysis sapphirina* would fall in synonymy with *Chrysis inaequalis* and the western subspecies would be named: Chrysis inaequalis ssp. taeniophrys Förster, 1853, which is the first available name. Moreover, if future examinations made with the help of molecular techniques will demonstrate that western and the eastern subspecies (*sensu* Linsenmaier) are separated and valid species, the valid name for *Chrysis inaequalis* in Europe would become *Chrysis taeniophrys* Förster, a name never used after the description given by Förster. In addition, the type of *Chrysis taeniophrys* Förster is lost, and we could not check that it is truly the first available name for the western form of *Chrysis inaequalis*. By designating a western European specimen, we keep the stability of name use. Therefore, the male specimen collected in Swtizerland at Roveredo on the 28^th^ of August 1948 by Linsenmaier (NML_ENT GBIF_Chr 00038702) is selected, housed in the Linsenmaier collection at the NMLS.

##### Current status.

*Chrysis inaequalis* Dahlbom, 1845.

#### Cleptes aurata

Taxon classificationAnimaliaHymenopteraChrysididae

Dahlbom, 1845

Cleptes aurata : Dahlbom 1845: 2, *nec* Panzer, 1798.

##### Type locality.

“*Bosfor*, *Hedenborg*”.

##### Remarks.

Móczár (1998b: 511) designated the neotype of *Cleptes aurata* Dahlbom on a female specimen collected by Houska in Palestina and deposited at the HNHM.

##### Current status.

*Cleptes dahlbomi* Semenov-Tian-Shanskij, 1909 (replacement name for *Cleptes aurata* Dahlbom, 1845).

### Specimens labelled as types but never described

In the general collection at the NHRS there is a specimen labelled: [J. Klapperich Sarekanda, 4100m 28.7.53, Gebirge Badakschan NO – Afghanistan] [*Chrysis badakschensis* n.sp ♀ Holotypus] <red label handwritten by Balthasar>. This species was never described by Balthasar and it belongs to the *Chrysis comparata* group, *analis* subgroup.

## Conclusions

The study of the type material by Dahlbom is fundamental to further knowledge on the European and western Palaearctic fauna. While studying his works, some interesting observations on types were found that were overlooked in recent revisions, probably because they were written in Latin. After reading Dahlbom’s main works (1845, 1854), we concluded that there is no correspondence between many descriptions and the current interpretation of the species. For this reason and in preparation of the volume on the Italian Fauna, a revisional work on the European types at the most important museums has been initiated by the first author (Rosa 2009; Paukkunen et al. 2014; Rosa and Xu 2015; Rosa et al. 2015), with multiple discoveries at different museums.

During the study of the type specimens housed in the NHRS, 72 types belonging to 53 taxa were examined. Some nomenclatural and taxonomic changes are proposed. Moreover, in contrast to the catalogue of the Chrysididae of the world (Kimsey and Bohart 1991), we found that two additional holotypes are deposited at the NHRS (*Chrysis equestris* Dahlbom, 1854 and *Omalus coriaceus* Dahlbom, 1850); three syntypes belonging to two species are deposited at the NHRS (*Chrysis manicata* Dahlbom, 1854 and *Chrysis soror* Dahlbom, 1854); and four holotypes and two syntypes are deposited at the NHRS and not at the MZLU or at the NMPC (*Chrysis elvira* Balthasar, 1957, *Chrysis klapperichi* Balthasar, 1957, *Chrysis nisseri* Dahlbom, 1845, *Hedychrum massaicum* Cameron, 1910, *Holopyga dohrni* Dahlbom, 1854).

## Supplementary Material

XML Treatment for Argochrysis albicornis

XML Treatment for Argochrysis armilla

XML Treatment for Argochrysis litura

XML Treatment for Ceratochrysis concava

XML Treatment for Ceratochrysis minata

XML Treatment for Chrysis bohemanni

XML Treatment for Chrysis ciscirtana

XML Treatment for Chrysis corusca

XML Treatment for Chrysis dalmanni

XML Treatment for Chrysis delicatula

XML Treatment for Chrysis diversa

XML Treatment for Chrysis elvira

XML Treatment for Chrysis equestris

XML Treatment for Chrysis erythromelas

XML Treatment for Chrysis imperialis

XML Treatment for Chrysis jugum

XML Treatment for Chrysis klapperichi

XML Treatment for Chrysis grohmanni ssp. krkiana

XML Treatment for Chrysis lateralis

XML Treatment for Chrysis lucifera

XML Treatment for Chrysis manicata

XML Treatment for Chrysis modica

XML Treatment for Chrysis nisseri

XML Treatment for Chrysis obsoleta

XML Treatment for Chrysis prominula

XML Treatment for Chrysis purpureifrons ssp. helleniensis

XML Treatment for Chrysis pyrrhina

XML Treatment for Chrysis rufiventris

XML Treatment for Chrysis schoenherri

XML Treatment for Chrysis scintillans

XML Treatment for Chrysis sinuata

XML Treatment for Chrysis sinuosa

XML Treatment for Chrysis soror

XML Treatment for Chrysis tenuimediata

XML Treatment for Chrysis violacuna

XML Treatment for Chrysis wahlbergi

XML Treatment for Chrysura candens

XML Treatment for Chrysura foveata

XML Treatment for Chrysura humboldti

XML Treatment for Chrysura sulcata

XML Treatment for Cleptes fasciata

XML Treatment for Cleptes sjostedti

XML Treatment for Cymura splendida

XML Treatment for Hedychrum massaicum

XML Treatment for Hexachrysis sjostedti

XML Treatment for Holopyga amoenula

XML Treatment for Holopyga dohrni

XML Treatment for Omalus coriaceus

XML Treatment for Pentachrysis kibonotoensis

XML Treatment for Platycelia ehrenbergi

XML Treatment for Stilbum hedenborgi

XML Treatment for Stilbum wesmaeli

XML Treatment for Stilbum westermanni

XML Treatment for Chrysis gloriosa

XML Treatment for Chrysis inaequalis

XML Treatment for Cleptes aurata
